# Development of Pectin-Coated Cationic Liposomes for Sustained Drug Release, Enhanced Serum Stability and Reduced Macrophage Uptake

**DOI:** 10.3390/pharmaceutics18101275

**Published:** 2026-10-08

**Authors:** Plamen Simeonov, Velislava Todorova, Tsvetelina Batsalova, Balik Dzhambazov, Stanislava Ivanova, Plamen Katsarov

**Affiliations:** 1 Department of Pharmaceutical Technology and Biopharmacy, Faculty of Pharmacy, Medical University of Plovdiv, 15A Vasil Aprilov blvd, 4002 Plovdiv, Bulgaria; plamen.simeonov@mu-plovdiv.bg; 2 Research Institute at Medical University of Plovdiv (RIMU), 4002 Plovdiv, Bulgaria; velislava.todorova@mu-plovdiv.bg (V.T.); stanislava.ivanova@mu-plovdiv.bg (S.I.); 3 Department of Pharmacognosy and Pharmaceutical Chemistry, Faculty of Pharmacy, Medical University of Plovdiv, 15A Vasil Aprilov blvd, 4002 Plovdiv, Bulgaria; 4 Department of Developmental Biology, Faculty of Biology, Paisii Hilendarski University of Plovdiv, 24 Tsar Assen Str., 4000 Plovdiv, Bulgaria; tsvetelina@uni-plovdiv.bg (T.B.); balik@uni-plovdiv.bg (B.D.)

**Keywords:** coated liposomes, pectin coating, sustained drug release, reduced macrophage uptake

## Abstract

**Background**: Cationic liposomes are promising carriers for anticancer agents but are often limited by rapid clearance by the mononuclear phagocyte system, poor serum stability, and insufficient control of drug release. This study aimed to develop and characterize pectin-coated, Ca^2+^-crosslinked cationic liposomes as a multifunctional delivery platform providing sustained release, enhanced serum stability, and reduced macrophage uptake while preserving the biological activity of an encapsulated anticancer compound. **Methods**: The optimal polysaccharide-to-liposome ratio was established by ζ-potential polyelectrolyte titration, and coating parameters were optimized using a Taguchi experimental design. Physicochemical characterization included dynamic light scattering (DLS), transmission electron microscopy (TEM), isothermal titration calorimetry (ITC), small-angle X-ray scattering (SAXS), and differential scanning calorimetry (DSC). The optimized formulations were further evaluated for drug release, serum stability, cellular uptake and cytotoxicity against A549 and HeLa cells using MTT and Neutral Red assays. **Results**: Polyelectrolyte titration identified a pectin-to-liposome saturation ratio of 3:1, and Taguchi optimization yielded a formulation with a drug retention efficiency of 99.34%. Pectin coating significantly reduced the initial burst release (37.9% at 1 h) and decreased cumulative drug release after 48 h (66.3%), consistent with a polymer relaxation-controlled release mechanism. The coated liposomes also exhibited enhanced serum stability and reduced macrophage uptake (up to ~73% lower in RAW264.7 cells), while maintaining uptake by non-phagocytic cells. The biological activity of the encapsulated model compound was preserved, with enhanced cancer-cell selectivity against A549 and HeLa cells demonstrated by both MTT and Neutral Red assays. **Conclusions**: Pectin-Ca^2+^ surface functionalization transforms cationic liposomes into a stable sustained-release delivery platform with reduced macrophage recognition and enhanced serum stability. These findings support the further development of this carrier as a versatile platform for the systemic delivery of anticancer agents.

## 1. Introduction

Natural compounds of plant origin have attracted significant interest as antitumor and chemopreventive agents, supported by epidemiological evidence linking regular consumption of fruits and vegetables with a reduced incidence of several cancers [1]. Among these, dietary polyphenols such as resveratrol, curcumin, and quercetin have been studied most extensively [2]. These polyphenols modulate multiple molecular targets, increasing the expression of pro-apoptotic proteins while decreasing that of anti-apoptotic ones, and act largely through their antioxidant capacity to eliminate reactive oxygen and nitrogen species, chelate metal ions, and modulate inflammatory signaling pathways [3]. Despite this therapeutic promise, the clinical translation of these compounds is constrained by some well-documented limitations. The natural polyphenols are characterized by low bioavailability and poor aqueous solubility, which can make it difficult to achieve the blood concentration required for the desired effect; their reduced in vivo bioavailability, owing to rapid metabolism, represents the major bottleneck to their use as anticancer therapeutics [4]. A promising solution lies in the structural modification of these compounds or in the development of novel delivery systems that can improve their solubility and bioavailability [5,6].

Xanthohumol, a prenylated chalcone from *Humulus lupulus*, structurally related to the polyphenols, has attracted considerable interest as a chemo preventive and antitumor agent owing to its activity against multiple oncogenic pathways [7]. Its translational potential is, however, constrained by poor aqueous solubility and limited bioavailability, which restrict the concentrations attainable at target tissues [8]. Encapsulation within different nano-carriers is an established strategy to overcome these limitations, improving the solubility of the substance, protecting the payload, and modulating its biodistribution [9].

Cationic liposomes are particularly attractive carriers for hydrophobic polyphenols because the positively charged bilayer promotes association with negatively charged cell membranes and favors intracellular delivery. Two well-recognized liabilities offset these advantages. First, a strongly positive surface charge promotes opsonization and rapid recognition by the mononuclear phagocyte system (MPS), shortening circulation time and diverting the carrier away from its intended target [10]. Second, conventional liposomes often exhibit a pronounced burst release that limits sustained exposure [11]. Surface modification with different polymers is a route to address both issues simultaneously, by shielding the surface and by imposing an additional diffusional barrier to the drug release. Apart from this, polymer coating can confer a broader set of benefits: it can improve the colloidal and mechanical stability of the vesicles, reduce premature drug leakage, protect the payload from enzymatic and chemical degradation, and prolong systemic circulation by limiting phagocytic uptake [12,13,14].

**Figure 1:**
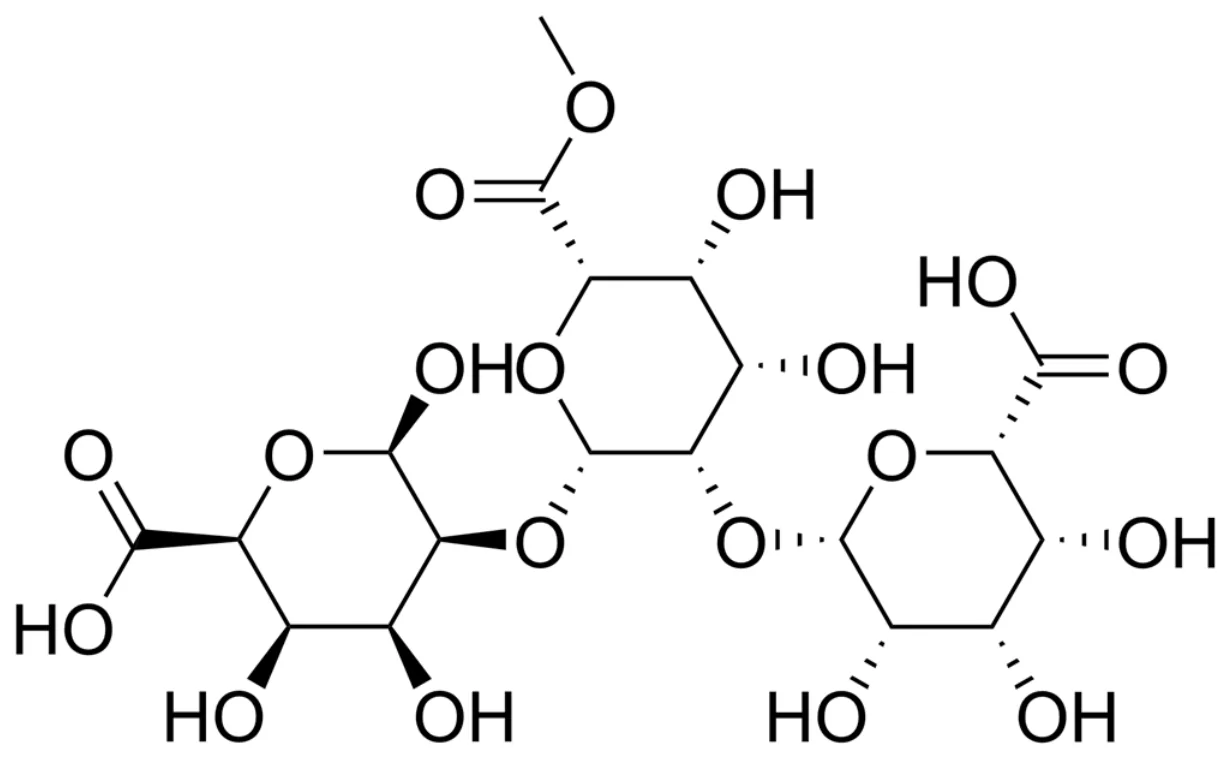
Representative segment of the pectin backbone, showing three α-(1→4)-linked D-galacturonic acid residues (drawn using ChemDraw, version 25.0.2.14, PerkinElmer).

Natural polysaccharides are a particularly compelling choice for liposomes coating materials, as they are biocompatible, biodegradable, and generally regarded as safe, while their abundant functional groups enable straightforward electrostatic or covalent attachment to the vesicle surface. In addition to these general advantages, individual polysaccharides offer intrinsic functional properties—such as mucoadhesion, tissue-specific targeting, and stimulus-responsiveness—without the need for further chemical modification [15,16]. Pectin, an anionic polysaccharide rich in galacturonic acid, can be considered as a suitable candidate for this purpose (Figure 1) [17]. Its dissociated carboxyl groups enable electrostatic adsorption onto cationic vesicles, and its capacity to form Ca^2+^-mediated “egg-box” junction zones allows the adsorbed layer to be reinforced into a coherent, crosslinked hydrogel shell. Such a shell may confer steric and mechanical stabilization, prolong drug release, and reduce non-specific recognition by phagocytes, while remaining biocompatible and biodegradable [18].

Therefore, the objective of the present study was to engineer pectin-coated, Ca^2+^-crosslinked cationic liposomes as an improved delivery platform for hydrophobic anticancer agents. Using xanthohumol as a model compound, we investigated the influence of pectin coating on the physicochemical properties of the liposomes, drug release behavior, serum stability, macrophage uptake, and in vitro biological activity.

## 2. Materials and Methods

### 2.1. Chemicals and Reagents

Xanthohumol (Mw 354.39 g/mol) was purchased from Cayman Chemicals (Ann Arbor, MI, USA), LIPOID PC 16:0/16:0 (1,2-Dipalmitoyl-*sn*-glycero-3-phosphocholine, DPPC) and LIPOID DOTAP-Cl (1,2-Dioleoyloxy-3-trimethylammonium-propane chloride, DOTAP) were purchased from Lipoid GmbH (Ludwigshafen, Germany). Cholesterol (from sheep wool, ≥92.5%), Pectin from citrus peel (galacturonic acid ≥ 74.0%, Mw 23–71 kDa), CaCl_2_ (Mw: 110.98 g/mol, ≥97.0%), FITC (Fluorescein 5(6)-isothiocyanate, Mw 389.38), DAPI (4′,6-Diamidino-2-phenylindole dihydrochloride) and Fetal Bovine Serum were purchased from Sigma-Aldrich (St. Louis, MO, USA). Trisodium citrate was purchased from Fillab (Plovdiv, Bulgaria). Ethanol (99.9%) was purchased from RaiChim (Plovidv, Bulgaria), acetonitrile, methanol (analytical grade), MTT (3-(4,5-dimethylthiazol-2-yl)-2,4-diphenyltetrazolium bromide) and Neutral Red were purchased from Merck KGaA (Darmstadt, Germany).

### 2.2. Preparation of Xanthohumol Loaded Liposomes

Xanthohumol-loaded liposomes were prepared by the ethanol injection method, as described in our previous work [19]. Briefly, DPPC, DOTAP, Cholesterol (7:1:2 mol/mol ratio), at total concentration of 1 mg/mL, and Xanthohumol (at Lipid phase: Xanthohumol 3:1 m/m ratio), were dissolved in 10 mL absolute ethanol. The resulting solution was heated above the phase transition temperature of the lipids (45 °C) and injected rapidly through 27 G needle into 100 mL ultrapure water, which was also equilibrated at the same temperature. The suspension was kept under magnetic stirring for a period of 1 h and then the residual ethanol was evaporated using the BUCHI RII Rotavapor rotary vacuum evaporator (BÜCHI Labortechnik AG, Flawil, Switzerland) under reduced pressure. After that, the liposomal suspension was stored at 4 °C, overnight, to allow the complete vesicle formation.

### 2.3. Surface Modification of the Liposomes

The formulated xanthohumol-loaded liposomes were subsequently modified with citrus pectin by employing the method of single-layer deposition, followed by cross-linking of the formed coating with CaCl_2_. The optimal polysaccharide:liposome ratio for the complete coverage of the vesicle surface, was determined by polyelectrolyte titration based on measurements of the surface charge density, or zeta-potential. A series of mixtures was prepared at varying polysaccharide:liposome weight ratios (*w*/*w*) at the following ratios: 1:15, 1:10, 1:5, 1:3, 1:1, 3:1, 5:1, 10:1, and 15:1. For each ratio, the defined volume of liposomes with a concentration of 1.0 mg/mL was added dropwise to a solution of pectin with a concentration of 1.0 mg/mL. This process was carried out under continuous magnetic stirring. The mixtures were then subjected to an incubation period of 60 min at ambient temperature. The ζ-potential of each mixture was measured, and the optimal ratio for each polysaccharide was defined as the point at which electrostatic saturation of the liposomal surface was reached (i.e., the ratio beyond which no further significant change in ζ-potential occurred).

Following the establishment of the optimal polysaccharide:liposome ratios, pectin was applied to the surface of the liposomes via single layer-deposition technique. Nine models were prepared using the L9 Taguchi orthogonal array. A defined amount of liposomes at a concentration of 1 mg/mL was added dropwise to a solution of polysaccharide with varying concentrations (0.5 mg/mL, 1.0 mg/mL, 2.0 mg/mL) at a fixed polysaccharide:liposomes ratio. The suspension was then incubated for 30 min, 60 min, or 120 min. Subsequently, a solution of CaCl_2_ was added at varying concentrations (1 mM, 5 mM, 10 mM), and the suspension was kept stirring for an additional 60 min to achieve complete equilibrium.

### 2.4. Liposomes Particle Average Size, Particle Size Distribution and ζ-Potential

The average hydrodynamic diameter, particle size distribution and ζ-potential of the formulated liposomes as well as the surface-modified liposomes were determined via dynamic and electrophoretic light scattering with a particle size analyser (Zetasizer UltraRed, Malvern Panalytical Ltd., Malvern, UK).

### 2.5. Entrapment Efficiency of Xanthohumol (EE)

The EE was determined for both the unmodified liposomes and the modified models through measurement of the incorporated xanthohumol. The separation of unentrapped xanthohumol was achieved through ultracentrifugation. An aliquot of each formulation was subjected to a centrifugal process at 30,070× *g* for 90 min at 4 °C using a Sigma 3-18KS centrifuge equipped with a fixed-angle rotor (Sigma Laborzentrifugen GmbH, Osterode am Harz, Germany). The pellet containing the vesicles was then subjected to an incubation with methanol and sonication for a period of 10 min. This procedure was undertaken to ensure complete lysis of the vesicles and complete solubilization of xanthohumol. The procedure described above was utilized for the determination of the drug retention efficiency after surface modification of the liposomes. Prior to the addition of methanol, 50 mM trisodium citrate was incorporated to chelate Ca^2+^. The mixture was then incubated for 30 min to facilitate the dissolution of the coating shell. Thereafter, the liposomes were subjected to ultracentrifugation under the aforementioned conditions. The resultant pellet was then incubated with methanol to solubilize the xanthohumol. The xanthohumol content was quantified by high-performance liquid chromatography (HPLC) using an LC40-PDA system (Shimadzu, Kyoto, Japan) equipped with a Shim-pack C18 column (150 × 4.6 mm, 3 μm; Shimadzu, Kyoto, Japan), following the analytical method described in our previous study [19]. The EE was calculated according to the following Equation (1):

*EE*% = *W_entrapped_*/*W_total_* × 100 (1)

where

W_entrapped_—amount of incorporated Xanthohumol
W_total_—total amount of Xanthohumol
For surface-modified liposomes, W_total_ was taken as the mass of xanthohumol entrapped in the optimal uncoated formulation prior to the coating step.

### 2.6. Small-Angle X-Ray Scattering (SAXS)

SAXS was used to characterize the nanoscale structure of the uncoated and pectin-coated liposomes. Measurements were performed on aqueous dispersions of the samples, recording the scattered X-ray intensity as a function of the scattering vector q; the scattering of the dispersion medium was recorded separately and subtracted from each sample. Data were collected on an Empyrean diffractometer (Malvern Panalytical, The Netherlands) equipped with a copper X-ray source (Cu Kα, λ = 1.5406 Å) operated at 45 kV and 40 mA, using a 4 × Ge (220) monochromator to obtain highly monochromatic incident radiation and a PIXcel3D detector, over the small-angle region 0.01–5° (2θ).

The background-subtracted scattering profiles were analyzed with the easySAXS software package (version 2.1, Malvern Panalytical, Almelo, The Netherlands). Structural parameters were derived by modelling the pair-distance distribution function (PDDF, p(r)) obtained through an indirect Fourier transformation of the experimental scattering data, from which the radius of gyration (Rg) and the maximum particle dimension (rmax/Dmax) were determined and the overall particle shape assessed. The quality of the fit was evaluated using the goodness-of-fit and stability (OSCILL) parameters provided by the software. The uncoated and pectin-coated formulations were compared based on the resulting p(r) profiles and the derived structural parameters.

### 2.7. Transmission Electron Microscopy (TEM)

The morphological characteristics of the unmodified as well as the pectin-modified liposomes were examined by transmission electron microscopy (Talos F200C Thermo Fisher Scientific, Waltham, MA, USA). Aliquots of the suspensions were deposited dropwise on a 200-mesh formvar/carbon-coated copper grids. The samples were air-dried overnight and imaged at 200 kV.

### 2.8. Differential Scanning Calorimetry (DSC)

DSC was used to characterize the thermal behavior of pure xanthohumol, uncoated xanthohumol-loaded liposomes, and pectin-coated xanthohumol-loaded liposomes. Analyses were performed on a DSC 250 calorimeter (TA Instruments, New Castle, DE, USA). Samples (1–5 mg) were weighed and sealed in hermetic aluminum pans and measured under an argon atmosphere from 20 to 90 °C at a heating rate of 2 °C min^−1^. Thermograms were processed with the TRIOS software (TA Instruments, New Castle, DE, USA), from which transition temperatures and enthalpies were determined.

### 2.9. Isothermal Titration Calorimetry (ITC)

ITC was used to probe the interaction between pectin and the cationic liposomes. Measurements were performed on a NanoAffinity ITC instrument (TA Instruments, New Castle, DE, USA) equipped with a gold cell (working volume 200 µL) at constant temperature. The titrant was injected sequentially into the cell containing the counterpart system, and the heat released upon each injection was recorded. Dilution controls (titrant into buffer and cell content into buffer) were run under identical conditions, and the integrated per-injection heats were compared with these controls.

### 2.10. In Vitro Drug Release Study

The in vitro release of xanthohumol from the formulated unmodified and pectin-modified liposomes models was evaluated using the dialysis bag method at pH 7.4 and at slightly acidic condition (pH 6.5). The release medium was composed of phosphate-buffered saline (PBS, pH 7.4 or pH 6.5) with 20% (*v*/*v*) methanol, a solvent that was incorporated to enhance the solubilization of the poorly water-soluble xanthohumol. The release medium was freshly prepared on the day of the experiment and equilibrated at 37 ± 0.5 °C prior to use. An aliquot of each formulation was placed inside a pre-soaked, 12-kDa MWCO dialysis membrane (Sigma, MWCO 12-kDa). The dialysis membrane was hydrated in ultra-pure water overnight and sealed with a plastic clamp. Each bag was then immersed in a beaker containing 200 mL of release medium and maintained under constant, gentle agitation on an electromagnetic stirrer at 150 rpm and 37.0 ± 0.5 °C. Aliquots of 2 mL were withdrawn at predetermined intervals and replaced with fresh medium. The quantity of xanthohumol released was determined by the method outlined in Section 2.5.

In order to verify that the dialysis membrane did not retain xanthohumol and thereby introduce bias into the measured release, a control experiment was conducted. In this experiment, 10 mL of free (non-encapsulated) xanthohumol solution, prepared in the release medium, was placed in the dialysis bag and dialyzed against 200 mL of the same medium under conditions identical to the release experiments. After 1 h, approximately 97% of the xanthohumol was recovered in the acceptor compartment, while approximately 2.8% remained in the donor compartment, resulting in a mass balance of ~99.8%. The membrane’s near-complete recovery indicates that it exhibited unobstructed permeability to xanthohumol and minimal adsorption or retention of the drug.

### 2.11. In Vitro Macrophage Uptake Assay

#### 2.11.1. Preparation of FITC Labelled Liposomes

For cellular uptake studies, fluorescently labelled liposomes were prepared with the same lipid composition, coating, and cross-linking as the xanthohumol-loaded formulations. These liposomes contained fluorescein isothiocyanate (FITC) encapsulated within the aqueous core as a fluorescent tracer. FITC was added to the aqueous hydration phase at an initial concentration of 0.1 mg/mL. The liposomes were formed by the ethanol injection method (Section 2.2) and coated and cross-linked as described. Unencapsulated free FITC was removed prior to all cellular experiments by ultracentrifugation under the same conditions used for the xanthohumol-loaded formulations (30,070× *g* for 90 min at 4 °C), and the amount of FITC incorporated was determined subsequently to be 0.052 mg/mL, corresponding to an encapsulation efficiency of approximately 52%. These FITC-labelled liposomes, matched in composition and surface modification to the drug-loaded formulations, were used to assess cellular uptake.

#### 2.11.2. Cell Lines and Culture Conditions

The biological evaluation of the xanthohumol (XH)-loaded liposomes comprised two sets of in vitro experiments: a cellular uptake study and an assessment of cytotoxicity and antitumor activity. The following human cell lines were used in these experiments: A549 (ATCC CCL-185™), derived from lung adenocarcinoma cells; HeLa (ATCC CCL-2™), derived from cervical adenocarcinoma cells; HT-29 (ATCC HTB-38), derived from colorectal adenocarcinoma cells; foreskin fibroblast cells—HFFC (CLS Cell Lines Service GmbH, Eppelheim, Germany). A murine macrophage line RAW264.7 (ATCC TIB-71) was used in the cellular uptake studies.

The adenocarcinoma cell lines and RAW264.7 macrophages were cultured in Dulbecco’s Modified Eagle Medium (DMEM) (Merck KGaA, Darmstadt, Germany) supplemented with 10% fetal bovine serum (FBS) (Merck KGaA, Darmstadt, Germany) and an antibiotic mixture containing 100 µg/mL streptomycin and 100 IU penicillin (Merck KGaA, Darmstadt, Germany). HFFC cells were grown in the same culture medium but with higher FBS concentration—15%. All cultures were maintained at 37 °C in a humidified incubator with a gas phase of 5% CO_2_ and 95% atmospheric air. Prior to the experiments, cells were seeded in 75 cm^2^ culture flasks (TPP, Trasadingen, Switzerland) and expanded under standard culture conditions until reaching 80% confluence.

#### 2.11.3. In Vitro Cell Uptake Assay

2 × 10^5^ cells/mL RAW264.7, A549 and HFFC suspensions were prepared and seeded onto a 96-well culture plate (TPP, Trasadingen, Switzerland). After 24 h, RAW264.7 cells were stimulated with 1 μg/mL lipopolysaccharide (LPS). The cells were cultured in LPS-containing medium for 24 h. Then, the cells were washed with Dulbecco’s Phosphate-Buffered Saline (DPBS) (Merck KGaA, Darmstadt, Germany) and treated with FITC-labelled liposomes for 1 h and 2 h. 24 h after seeding HFFC and A549 cells were incubated for 1 h and 2 h in medium containing 50 µg/mL FITC-labelled liposomes. At the end of the incubation periods with FITC-loaded liposomes, all cell types were washed three times with DPBS, and the cellular liposome uptake was analyzed based on fluorescence signal detection (excitation 495 nm/emission 525 nm) using a SpectraMax i3x spectrophotometer (Molecular Devices, San Jose, CA, USA).

To determine the amount of fluorescent dye released by the liposomes during cell culture, the culture medium was collected after incubating the cells with FITC-labeled liposomes. The samples were then centrifuged at 13,400 rpm for 1 h to pellet intact liposomes and cellular debris, and the resulting supernatant was analyzed. The concentration of FITC in the supernatant was accessed using a SpectraMax i3x spectrophotometer (Molecular Devices, San Jose, CA, USA) and the results were compared with a reference FITC standard in two-fold serial dilutions (concentration range 100–0.00625 µg/mL) included in the assay. During the 1 h and 2 h incubation of LPS-stimulated RAW264.7 cells in medium containing FITC-labeled liposomes the concentration of released FITC ranged from 0.010 to 0.035 µg/mL.

For fluorescence microscopy analyses, the method described by Wang et al. [20] was used with some modifications. RAW264.7 cells were seeded onto sterile slides at a concentration of 2 × 10^5^ cells/mL, incubated for 24 h, and then stimulated with 1 μg/mL LPS for 24 h. Subsequently, the cells were washed with DPBS and treated with FITC-labelled liposomes for 2 h. Afterward, the cells were washed three times with DPBS and fixed with a 4% paraformaldehyde solution for 20 min at room temperature. Then, the cells were washed twice with DPBS and incubated in the dark with a 0.75 µg/mL DAPI solution for 10 min at room temperature. The cells were then washed again with DPBS and observed using a Leica DM1000 LED epifluorescence microscope equipped with a camera (Leica DM 2000 LED, Leica Microsystems, Wetzlar, Germany) and an I3 filter (Leica Microsystems, Wetzlar, Germany). Leica Application Suite (LAS X 5.0.2) software was used to document and analyse the images obtained.

### 2.12. In Vitro Cytotoxicity and Antitumor Activity Assays

Two types of in vitro cytotoxicity assays were performed: MTT assays, which measure the reduction of 3-(4,5-dimethylthiazol-2-yl)-2,4-diphenyltetrazolium bromide (MTT) by metabolically active cells [21], and the NR assays, which are based on the uptake of neutral red (NR) dye into the lysosomes of viable cells [22]. The experiments were carried out following a procedure similar to a previously described one [23]. Briefly, cell suspensions with a concentration of 1 × 10^5^ cells/mL were prepared and seeded into 96-well plates (TPP, Trasadingen, Switzerland) at 100 µL/well, and cultured under standard conditions for 24 h. The cells were then treated with various concentrations of liposomes (1, 6.25, 12.5, 25, 50 µg/mL) for 24, 48, and 72 h. As controls for comparison with the effect of the liposomes, xanthohumol solution was used and analyzed at the same concentrations as the test samples. A control with non-treated cells cultured for the same test-period in standard growth medium was included in all assays.

At the end of each incubation period (24, 48, and 72 h), MTT or NR solution was added to the cells to a final concentration of 0.5 mg/mL or 0.005 mg/mL, respectively. The cultures were incubated for 2 h in the dark at 37 °C, 5% CO_2_, and high humidity. The culture medium was then removed. For the MTT assay, the cultures were washed with D-PBS (Merck KGaA, Darmstadt, Germany), after which the formazan accumulated in the cells was dissolved by adding 100 µL/well dimethyl sulfoxide (DMSO) and incubating at room temperature for 15 min under continuous gentle shaking. Absorbance was then measured at 570 nm using a SpectraMax i3x spectrophotometer (Molecular Devices, San Jose, CA, USA). For the NR assay, the dye accumulated in the cells was extracted with 100 µL/well aqueous solution of ethanol and acetic acid (50%:1%) for 15 min under continuous gentle shaking, and absorbance was subsequently measured at 540 nm using SpectraMax i3x spectrophotometer (Molecular Devices, San Jose, CA, USA). All samples were assayed in triplicates. The results are expressed as the percentage inhibition of cell viability or metabolic activity, calculated based on the absorbance units recorded for treated cells versus cells cultured under standard conditions without the addition of test sample. The liposome and XH concentration inhibiting the metabolic activity or viability of 50% of the cells (IC_50_) was determined for the 48 h test period.

### 2.13. Serum Stability Study

To complement the macrophage uptake study, the colloidal stability of the uncoated and polysaccharide-coated liposomes was assessed under simulated physiological conditions. Each formulation was diluted in 10% (*v*/*v*) FBS in PBS (pH 7.4) to a final lipid concentration of 0.08 mg/mL. Samples were transferred to sealed polypropylene microcentrifuge tubes and incubated statically, without agitation, at 37.2 °C for 48 h. A serum-only control (10% (*v*/*v*) FBS in PBS containing no liposomes) was prepared and incubated in parallel under identical conditions and was measured at both time points to establish the scattering contribution of the serum proteins themselves and of any protein aggregates formed during the incubation. Dynamic light scattering and electrophoretic light scattering techniques were used to monitor the changes in hydrodynamic diameter, polydispersity, surface chare and intensity autocorrelation functions.

### 2.14. Statistical Analysis

Statistical analysis of the factorial design, as well as the Taguchi design was performed using Minitab^®^ Statistical Software version 21.1 (Minitab LLC, State College, PA, USA; 2023). For the polyelectrolyte titration, a one-way analysis of variance (ANOVA) was performed on the ζ-potential data for all polysaccharide-to-liposome ratios, followed by Tukey’s honestly significant difference (HSD) post hoc test for pairwise comparisons. For in vitro cell uptake assays analyses StatView software (version 5.0) (SAS Institute, Cary, NC, USA) was used to apply analysis of variance (ANOVA). Statistically significant differences between the samples were determined using Fisher’s PLSD test. Lower than 0.05 *p*-values were considered as statistically significant. Unless otherwise stated, all experiments were conducted as three independent biological replicates, and the results are presented as the mean ± standard deviation (SD).

## 3. Results and Discussion

### 3.1. Formulation of Drug-Loaded Liposomes

The unmodified cationic liposomes used as the substrate for pectin modification were developed and optimized in our previous study [19], in which a 2^3^ + 1 full factorial design was employed to examine the influence of lipid concentration, DPPC:DOTAP:cholesterol ratio and lipid phase:xanthohumol ratio on vesicle size, ζ-potential and entrapment efficiency. The selected model combined a sufficiently small vesicle size for subsequent surface functionalization with a high positive surface charge suitable for electrostatic polysaccharide deposition and an acceptable entrapment efficiency, yielding an average diameter of 163 ± 2 nm, a ζ-potential of +47.6 ± 0.8 mV and an entrapment efficiency of 56.7 ± 0.3%. This formulation was used throughout the present work, and its properties are reproduced here only insofar as they are required to interpret the behavior of the pectin-modified vesicles.

### 3.2. Pectin-Coating of the Drug-Loaded Liposomes

#### 3.2.1. ζ-Potential Based Polyelectrolyte Titration

To establish the optimal polysaccharide-to-liposomes ratio and the degree of which the liposomes’ surface becomes completely saturated with pectin, a polyelectrolyte titration method was employed, as previously described by Correa et al. (2019) with slight modifications [24]. Different Pectin:Liposome ratios ranging from 15:1 to 1:15 were prepared. The statistical analysis of the ζ-potential values was performed using a One-way Analysis of variance (ANOVA), followed by a Tukey’s HSD (Honest Significant Difference) post hoc test. This approach facilitated the undertaking of two-way comparisons between the mean electrophoretic potential values of varying ratios. The statistical significance of the findings was determined by establishing a probability value of *p* < 0.05. The data is presented as the mean ± standard deviation (n = 3).

**Figure 2:**
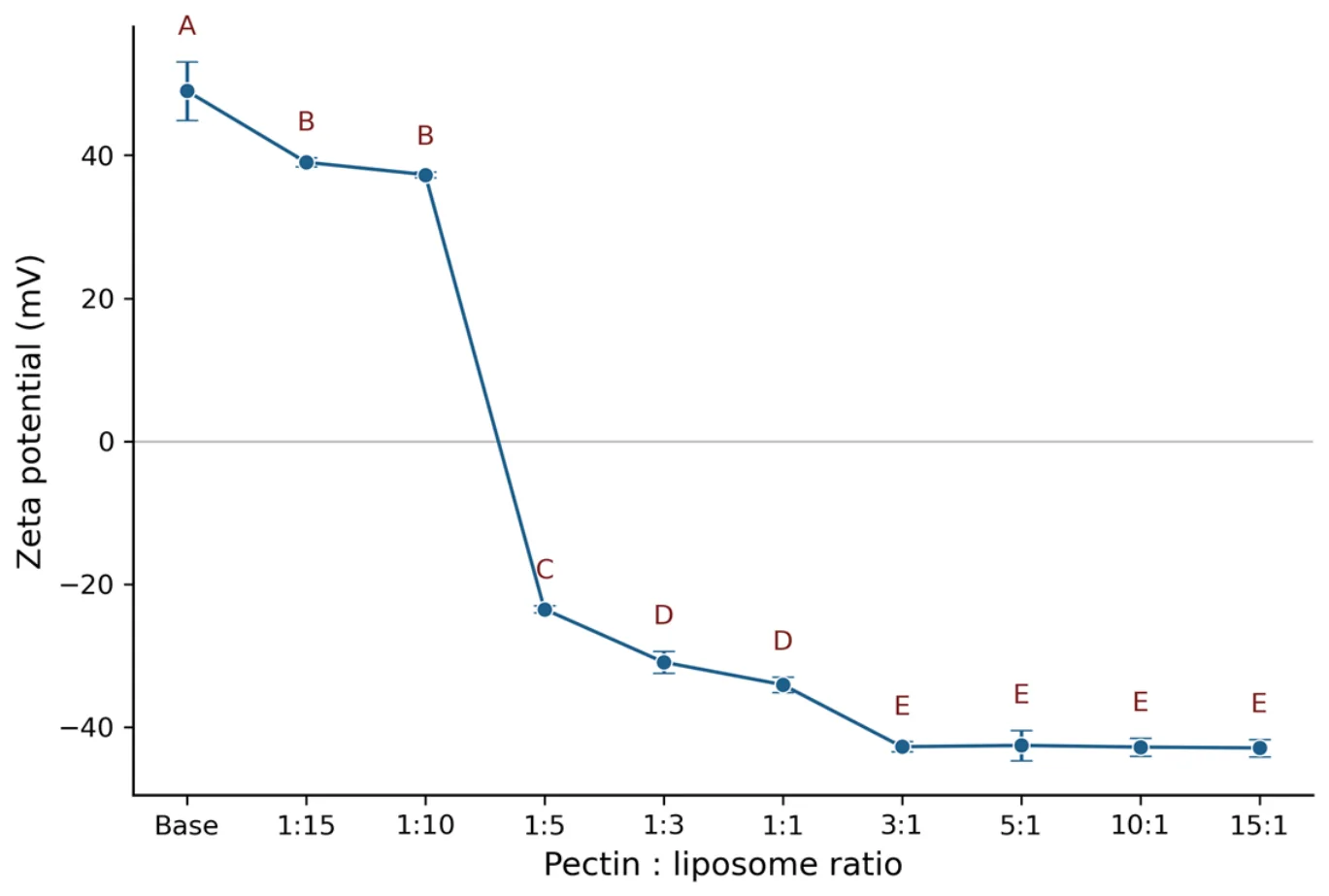
Interval plots of the ζ-potential, obtained from the polyelectrolyte titration. The letters represent statistical groups obtained by Tukey’s HSD post hoc test (Means that do not share a letter are significantly different).

One-way ANOVA revealed a significant effect of pectin:liposomes ratio on the ζ-potential (F value = 1027.32, *p* < 0.001, R^2^ = 0.9978). Post hoc analysis using Tukey’s HSD identified 5 distinct statistical groups (A–E) for the whole set of experiments (Figure 2). As seen from the data, the uncoated vesicles showed ζ-potential value of +47.57 mV (group A). After the initial introduction of the polysaccharide, at pectin:liposomes ratios of 1:10 and 1:5, a significant drop in the ζ-potential was observed (Group B); however, the surface charge remained positive, indicating that the liposomal surface is still dominating the overall net charge of the structure. For a polymer:liposomes ratio of 1:3, the ζ-potential crossed the isoelectric point with a sharp reversal of the surface charge (−23.5 mV). At a pectin:liposomes ratio of 3:1, a plateau was reached, showing that all results afterwards are indistinguishable sharing the same statistical grouping (group E).

The results from the experiment indicated that a pectin:liposomes ratio of 3:1 can be stated as the saturation point or the minimum ratio needed for surface coverage. From that point beyond, adding more polymer gives no significant change and may create potential interference with downstream characterization.

#### 3.2.2. Taguchi Experimental Design

Following the evaluation of the results from the polyelectrolyte titration, a Taguchi experimental design was implemented to establish the optimal setting for the preparation of pectin-coated liposomes, stabilized by cross-linking the surface modification. The design was utilized to assess the influence of the pectin concentration, the concentration of CaCl_2_, and the time of incubation on the hydrodynamic diameter, ζ-potential and the polydispersity index (PDI). Table 1 presents the design matrix and the determined parameters measured as mentioned above.

Influence of the variables on the average size of the modified liposomes.

The S/N ratio analysis identified that polysaccharide concentration is the primary determinant of the average size of the pectin-modified liposomes (Table S1), with ∆-value of 3.34, showing a monotonic increase throughout the different levels of variation, indicating that the average size of the formulated vesicles increased consistently with the increase in the concentration of the polysaccharide. The regression analysis (Table S2) identified that both the linear (F-value 1313.72, *p* < 0.001) as well as the quadratic effect (F-value of 81.71, *p* < 0.001) of pectin concentration had a statistically significant effect on the average size, demonstrating a J-shape curve effect. Since the pectin:liposomes ratio was fixed and as the concentration of the polysaccharide is increased, the concentration of liposomes increases proportionally, and the mechanistic explanation of the observed effect is probably due to the fact that as the concentrations increase, relative to the volume of the media, the inter-particle distance decreases proportionally. This probably leads to interparticle bridging, as one polymer chain can simultaneously adsorb onto two adjacent liposomes [24]. This effect explains the accelerating quadratic effect at high concentrations, as the system crosses a critical concentration threshold, where bridging becomes highly probable and dominating.

Data analysis defined CaCl_2_ as the second most dominant factor, which influences the average size of the formulated vesicles with ∆-value of 1.74. It is evident that the effect of the CaCl_2_ concentration is not as strong as the influence of the pectin concentration, which is also confirmed by the regression analysis, showing an F-value of 318.15 for its linear effect, which is almost 4 times lower than the corresponding parameter of the polysaccharide concentration, and 67.66 for its quadratic effect both of them determined to be statistically significant (*p* < 0.001). The positive linear coefficient (+34.70) shows a trend for an overall increase in the average size as the concentration of CaCl_2_ increases from 1 mM to 5 mM, which is probably due to the formation of a coherent gel-like shell around the liposomes [18], adding measurable physical thickness. However, the negative quadratic coefficient (−27.96) demonstrates that beyond this point the average size decreases, probably due to the contraction of the polymeric network and the shell becoming more compact [25], partially counteracting the size increase.

The conducted statistical analysis outlined the incubation time as the factor with the least impact on the average size (∆-value of 1.18). Despite that, both its linear and quadratic effects proved to be statistically significant (*p* < 0.005 for both effects); however, it is evident that the linear effect has an overall much stronger impact on the average size (F-value 200.04) compared to its quadratic effect (F-value of 11.70). As shown in Table S2, the negative linear coefficient (−27.36), shows that the average size tends to decrease as the incubation time gets longer, although the positive coefficient of the quadratic effects (+13.13), determines that the effect is not a straight line but rather slows down after 60 min. This can be explained by the fact that at 60 min the polysaccharide chains begin to relax and reorganize toward lower-energy conformations, approaching thermodynamic equilibrium [26,27]. In comparison, at 30 min, the rapidly adsorbed pectin chains set onto kinetically trapped conformation, producing loose, hydrodynamically large corona [28]. As already mentioned, the positive quadratic effect proves that the compaction slows at 120 min as equilibrium is approached, consistently with Langmuir-type adsorption kinetics [24].

Influence of the variables on the ζ-potential of the modified liposomes.

As seen in Table S3, the Taguchi analysis identified the concentration of CaCl_2_ with the highest effect on the ζ-potential with ∆-value of 7.47, compared to the other factors, which showed ∆-value of 0.90 (Incubation time) and 0.46 (Polysaccharide concentration). This shows that the CaCl_2_ is practically the primary governing factor of the ζ-potential. The data shows that, overall, the ζ-potential value drops as more Ca^2+^ ions are introduced to the system. This effect might be due to the formation of the so called “egg-box” conformation between Ca^2+^ and -COO^-^ groups, which at low concentration of CaCl_2_ (1 mM) remain mainly unbound and fully ionized, leading to a maximum negative surface charge density and high ζ-potential value, respectively [29]. It should be noted that at this concentration, the pectin coating is loosely crosslinked and chains remain mobile and extended [30]. Furthermore, as the concentration of CaCl_2_ is increased, Ca^2+^ begins coordinating adjacent pectin chain segments in the “egg-box” model and since it bridges two -COO^-^ groups simultaneously, it leads to a steep charge-reducing effect. As noted by the positive quadratic effect (Table S4), at high concentrations (10 mM) the Ca^2+^ encounters diminishing availability of free binding sites and thus the rate of charge reduction slows markedly, approaching system charge saturation and possibly accumulation of Ca^2+^ at the interface [31].

As mentioned already, in contrast to the influence of the concentration of CaCl_2_, the other two parameters affected the surface charge to a sufficiently lower degree, although both linear effects of polysaccharide concentration and incubation time show statistical significance (*p* < 0.01). As determined by the regression analysis, both linear parameters have negative coefficients (−0.974 for polysaccharide concentration and −1.298 for incubation time), which means that increasing both of them will lead to an overall decrease in the ζ-potential; however, their F-values are more than 30-times lower than the F-value of the CaCl_2_ concentration, proving that their effects on the ζ-potential can be considered negligible.

Influence of the variables on the polydispersity index of the modified liposomes.

The statistical data shows that the factor with the most significant effect on the PDI is the incubation time (∆-value of 2.89) (Table S5). The other two parameters show similar ∆-values to the one of the incubation time (2.27) for polysaccharide concentration and 1.96 for CaCl_2_), meaning that the three parameters affect the PDI almost equally. The regression analysis (Table S6) indicates a tendency for an overall decrease in the PDI as the incubation time is extended. At 30 min, most of the results show PDI above 0.25, which might be because pectin chains adsorb onto different liposomes at different rates, that depends on the local concentration gradients, chain length polydispersity within the pectin sample and stochastic differences in surface accessibility [26,32]. As a result, some liposomes accumulate thick, multilayer coatings, while others remain sparsely covered, which directly translates into a broad size distribution. Furthermore, at short times pectin chains have not yet committed to adsorbing onto a single liposome—chains with segments adsorbed on two adjacent liposomes act as transient bridges, creating a subpopulation of loosely bridged dimers and trimers coexisting alongside single coated particles [33]. Moreover, when Ca^2+^ is introduced early at this stage, it crosslinks and locks in these heterogeneous conformations, resulting in a highly polydisperse population, hence the lower S/N ratio at 30 min. As the incubation time is increased, sufficient time has elapsed for the pectin chains to redistribute across the liposomal surface and for the transiently bridged particles to dissociate and re-equilibrate, as the system approaches its equilibrium state. As seen by the positive quadratic coefficient (+0.05), this effect tends to slow down as the time moves on and practically the response at 60 and 120 min is almost equal, which is also confirmed by the nearly identical S/N ratios (14.08 and 14.14).

The polysaccharide concentration was identified as the second most impactful parameter over the PDI, with ∆-value of 2.27. The subsequent regression analysis showed that only its linear effect exerts a statistically significant effect (*p* < 0.001), but its F-value (18.80) is almost 2-times lower than that of the incubation time, meaning that the impact on the PDI was much weaker. The positive coefficient indicates that as the concentration of the polysaccharide (and respectively the liposomes) is increased, the PDI tends to grow larger. It can be explained with the reduced inter-particle distance, due to the higher particle density. This leads to an increased collision frequency between partially coated liposomes during the process of coating, proportionally increasing the probability of permanent bridging events [34]. This explains the linear behavior of the effect, simply as the number of the particles increases, the chance of occurrence of more encounters increases as well, leading to more heterogeneous aggregates.

The concentration of CaCl_2_ was determined to be the least impactful parameter (∆-value of 1.96). Similar to the polysaccharide concentration, only the linear effect exerts statistical significance (*p* < 0.005). The F-value (11.72) of the parameter is even lower, confirming the relatively low impact on the PDI. Nevertheless, its positive coefficient shows that as the concentration of CaCl_2_ increases, the PDI tends to increase as well, which might be due to the inter-particle crosslinking, predominantly occurring at high concentration of Ca^2+^.

#### 3.2.3. Optimization of the Pectin-Coating Results

The optimization of the obtained data aimed to identify the combination of parameters that provide minimal particle size while maintaining narrow and homogeneous size distribution, as well as sufficiently high negative ζ-potential. As presented above, the polysaccharide concentration affects the average size most significantly, leading to its massive increase as the concentration is increased, while affecting the PDI and ζ-potential to a significantly lesser impact. Thus, level 1 (0.5 mg/mL) was selected as the most appropriate level for the parameter. Regarding the concentration of CaCl_2_, data shows that the average size increases at 5 mM CaCl_2_ compared to 1 mM, apart from this the ζ-potential drops in the same range. However, it should be taken into account that these effects most likely occur due to the formation an “egg-box” shell on the surface of the liposomes, which sterically stabilizes the liposomes, despite the drop in the electrostatic charge of the particles [35]. Additionally, at 5 mM CaCl_2_ the PDI shows its lowest values (~0.160), which also confirms the formation of structurally coherent shell and indicates that the particle size increase is not due to particle agglomeration [36]. Considering all of this, 5 mM of CaCl_2_ was selected as the optimal level for the formulation. Finally, regarding the incubation time, it showed a tendency to a decrease in the average size and decrease in the PDI as it increased, while it practically had small effect on the ζ-potential. Although 120 min yielded marginally smaller particle size and PDI, the change of the parameters in both cases was considerably smaller compared to the change from 30 to 60 min. Based on practical efficiency and multi-response optimality, 60 min was selected as the most optimal time of incubation. The formulated optimal model (0.5 mg/mL polysaccharide concentration, 5 mM CaCl_2_ and 60 min incubation time) fully corresponds to the already developed model PEC2. The drug retention efficiency of the selected model was determined as well and was found to be 99.34% ± 0.02, which proved that the whole amount of Xanthohumol was retained after the process of surface modification.

### 3.3. Physicochemical Characterization of the Coated Liposomes

#### 3.3.1. ITC Analysis

Isothermal titration calorimetry (ITC) was used to characterize the energetics of the pectin–liposome interaction. Given that the liposomes carried a pronounced positive surface charge, conferred by the quaternary ammonium headgroups of the cationic lipid DOTAP [37], and that pectin behaves as a weak anionic polyelectrolyte by virtue of the dissociated carboxyl groups of its galacturonic acid residues [30], a strong electrostatically mediated interaction between the two species was hypothesized [38]. ITC was therefore employed to verify the existence of this interaction and to also confirm the stoichiometric ratio at which the liposomal surface becomes fully coated as determined previously by the polyelectrolyte titration.

**Figure 3:**
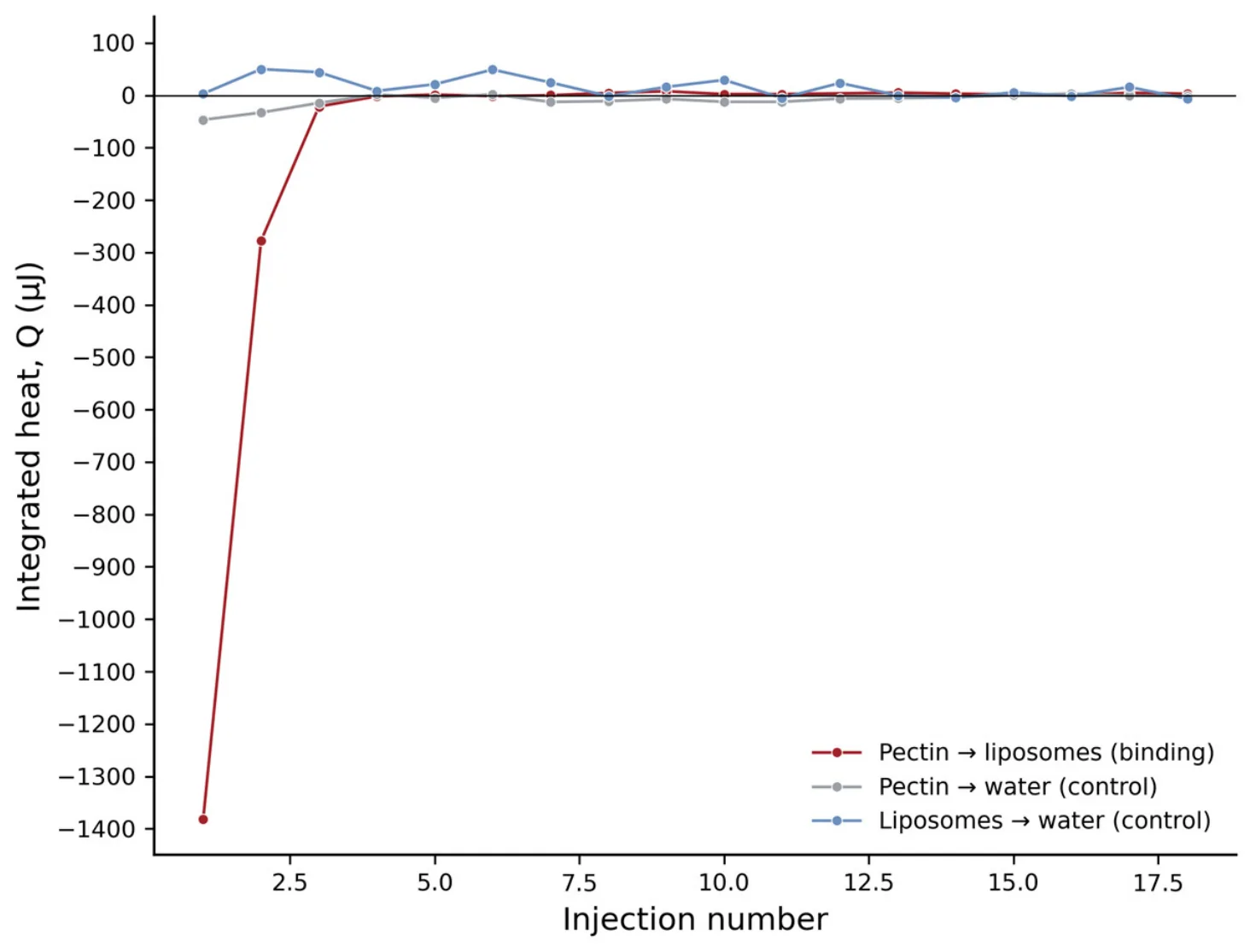
Isothermal titration calorimetry of the pectin–liposome interaction. Integrated heat per injection for pectin titrated into the liposome dispersion (binding), compared with the pectin-into-water and liposome-into-water dilution controls.

Two dilution controls (pectin into ultra-pure water and liposomes into ultra-pure water) were performed to distinguish genuine interaction heat from the heats of dilution and hydration of the individual components (Figure 3). Titration of pectin into water produced a moderate, exothermic response, largest on the initial injections and decaying as the cell is filled with polysaccharide; this profile is characteristic of the dilution and de-aggregation of a concentrated polysaccharide, in which entangled chains disperse and hydrate, forming favourable hydrogen bonds with water [39]. Titration of the liposomes into water produced a weaker, endothermic response, consistent with dilution-driven reorganisation of the lipid membrane and the release of structured water. In neither control was a sigmoidal binding transition observed, and the controls were therefore treated as descriptions of the respective dilution processes rather than as binding events; the pectin dilution heats were subtracted from the titration of pectin into liposomes to isolate the net heat of interaction.

Titration of pectin into the cationic liposome dispersion produced substantial exothermic heats during the initial injections, markedly exceeding any signal recorded in the dilution controls and decaying rapidly to the level of the dilution baseline within the first few injections. This profile is the calorimetric signature of a strong, high-affinity and essentially stoichiometric association: each early increment of pectin was bound almost completely by the available surface until coverage approached saturation, beyond which only the heat of dilution remained. The pronounced exothermicity is consistent with direct electrostatic ion-pairing between the anionic galacturonate carboxylate groups of pectin and the cationic trimethylammonium headgroups of DOTAP at the positively charged vesicle surface [30]. This interpretation is independently corroborated by the polyelectrolyte titration, in which coating reversed the surface charge from +47.57 mV to a net negative value, demonstrating that pectin adsorbs in sufficient quantity to neutralise and overcompensate the cationic surface—a behaviour explicable only by an electrostatically driven polyelectrolyte–membrane association. The termodynamic parameters of the interaction are summarized in Table S7. The observed charge reversal and particle size increase are consistent with recent literature on pectin functionalized vesicular systems. Lutta et al. (2025) similarly reported a complete surface charge reversal and comparable hydrodynamic size increase after the deposition of the pectin layer o nthe cationic liposomal surface [38].

#### 3.3.2. SAXS Analysis

**Figure 4:**
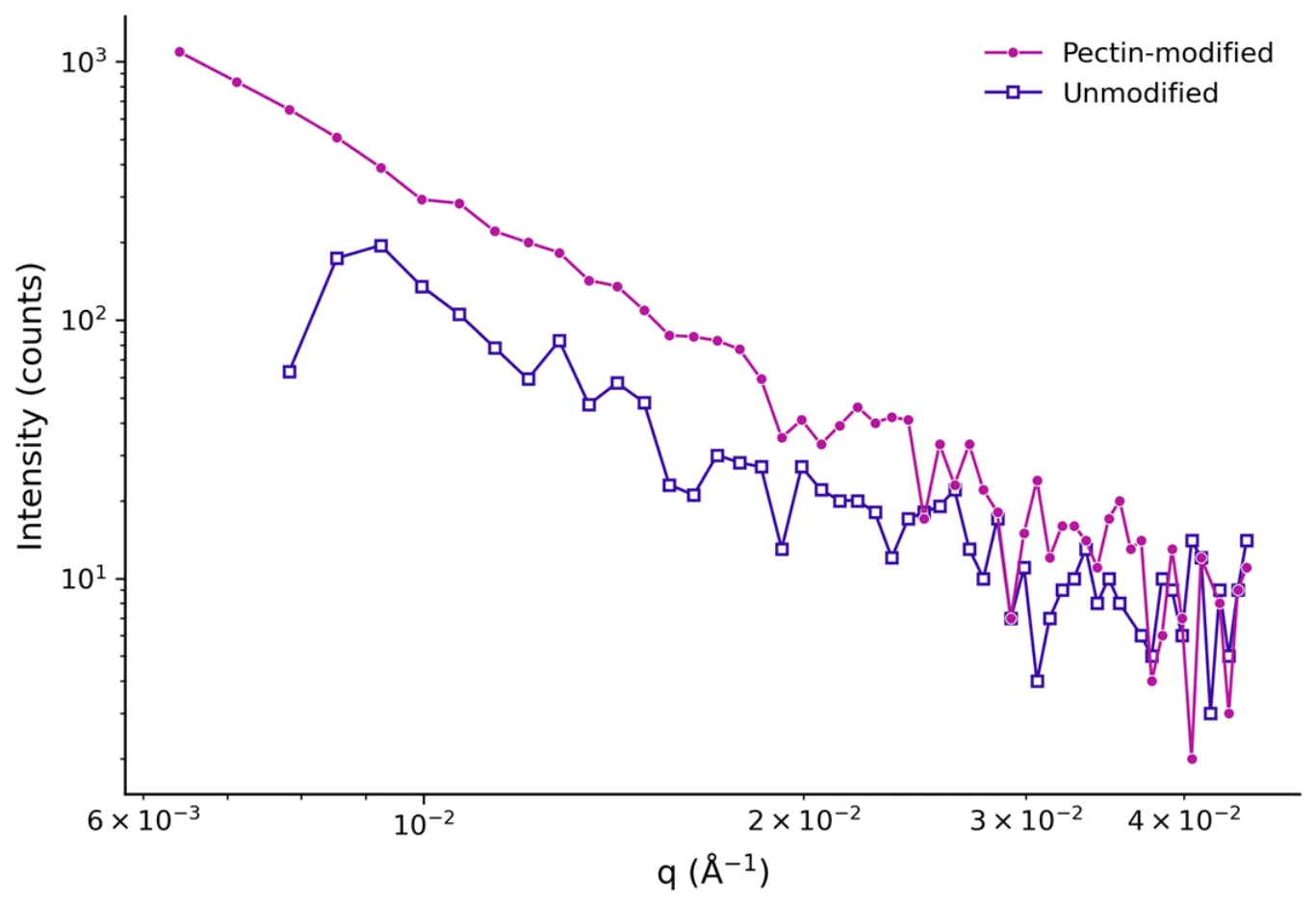
Background-subtracted SAXS profiles of unmodified and pectin-modified liposomes.

Small-angle X-ray scattering (SAXS) measurements were performed to elucidate the nanoscale architecture of liposomes. The pair distance distribution function (p(r)), derived from experimental scattering data via an indirect Fourier transformation, exhibited a profile characteristic of globular, hollow particles for the unmodified vesicles (Figure 4). This confirmed the successful formation of closed vesicular structures. The resulting radius of gyration (R_g_) was determined to be 304.3 Å (30.4 nm). The goodness of fit, as indicated by a value of 0.750, and the OSCILL parameter, with a value of 2.080, are indicative of satisfactory agreement between the experimental data and the theoretical model (Figure S1). The R_g_/r_max_ ratio of approximately 0.40 is consistent with hollow spherical geometries, approaching the theoretical value of ~0.387 expected for thin-shelled spherical particles. This finding supports the hypothesis that the xanthohumol-loaded DPPC:DOTAP:CHL formulation undergoes effective self-assembly into vesicular structures within an aqueous suspension, with the hydrophobic xanthohumol molecules potentially intercalated within the hydrocarbon core of the lipid bilayer, as has been documented for other polyphenolic compounds within similar cationic liposomal systems [40].

A direct comparison of SAXS-derived parameters between the uncoated and pectin-coated liposome formulations reveals profound structural changes induced by the polysaccharide coating process. Most notably, both the radius of gyration and the maximum particle dimension exhibited a substantial decrease following pectin coating and Ca^2+^ crosslinking. The R_g_ decreased from 30.4 nm to 19.2 nm (36.8% reduction), while r_max_ decreased from 76.5 nm to 50.2 nm (34.4% reduction). The quality of fit exhibited a marginal improvement, with an increase in goodness of fit from 0.750 to 0.798. Concurrently, the OSCILL parameter diminished from 2.080 to 1.313, indicating a homogeneous particle population following the coating process. The overall shape of p(r) retained the essential features expected for globular particles—zero at the origin, a single broad maximum, and return to baseline at r_max—confirming that the coated formulation still comprises closed vesicular structures rather than disrupted lipid aggregates or micellar phases.

In coated vesicular systems, a hydrated polysaccharide shell is highly solvated and composed of light atoms, giving it an electron density very close to that of the aqueous medium [41]. As a consequence, such a shell is effectively “invisible” to the X-ray beam—a phenomenon known as contrast masking—so that SAXS reports predominantly on the electron-dense lipid bilayer rather than on the full, coating-inclusive particle. This explains the apparent discrepancy with dynamic light scattering, which detects the complete hydrodynamic particle including the hydrated corona and therefore records a larger diameter for the coated liposomes [42,43]. While the contrast-masking effect of the pectin shell explains why SAXS does not detect the full coated particle dimension, the reduction in the SAXS-derived r_max_ (from 76.5 nm to 50.2 nm) suggests that additional structural changes occur within the lipid bilayer domain during the coating process. This can be due to a pectin induced bilayer compression or lipid rearrangement at the membrane interface. Electrostatic interactions between the anionic galacturonic acid groups of pectin and the cationic trimethylammonium headgroups of DOTAP probably leads to localized lipid packing changes, reducing the effective bilayer thickness or altering the membrane curvature in a manner that decreases the overall vesicle dimension. Such membrane-remodeling effects have been documented for other polyelectrolyte–lipid interactions in layer-by-layer coated liposome systems and may contribute to the SAXS-observed contraction of the lipid-enclosed volume [44]. The osmotic dehydration during the coating process might also contribute to the apparent reduction in the bilayer size; however, definitive separation of the two contributing factors is an objective for a future study.

#### 3.3.3. TEM Analysis

**Figure 5:**
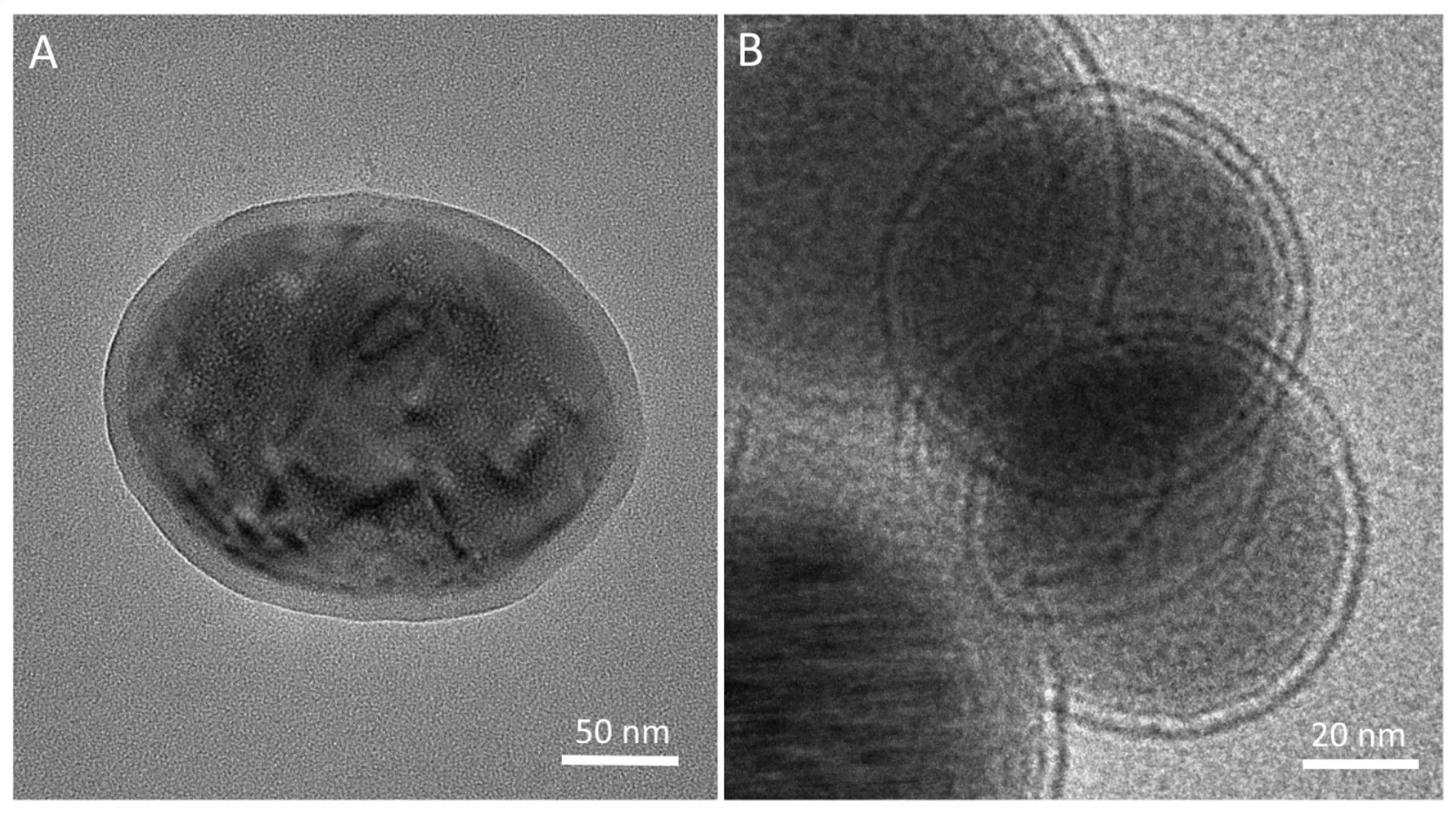
Transmission electron micrographs of unmodified (**A**) and pectin-modified (**B**) xanthohumol-loaded liposomes.

The TEM micrograph (Figure 5) confirmed the successful formation of well-defined liposomal vesicles with a predominantly spherical morphology and a continuous lipid bilayer.

As expected, the apparent dimensions observed by TEM should be interpreted qualitatively, since dehydration during sample preparation may induce partial vesicle collapse and structural shrinkage. Following surface modification, the pectin-coated liposomes exhibited a distinct layered morphology, providing direct visual evidence for the successful deposition of the Ca^2+^-crosslinked pectin shell. The coated vesicles displayed a clearly distinguishable outer electron-transparent layer surrounding the lipid core, consistent with the formation of a polymeric coating. The apparent reduction in vesicle dimensions compared with the uncoated liposomes is attributed to dehydration-induced contraction of the hydrated pectin hydrogel during air-drying, whereas DLS measures the fully hydrated particles in suspension. Thus, TEM primarily confirms the successful surface functionalization and structural integrity of the coated liposomes, complementing the physicochemical information obtained by DLS and SAXS.

#### 3.3.4. DSC Analysis

Differential scanning calorimetry (DSC) was employed to characterize the thermal behavior of pure xanthohumol, xanthohumol-loaded uncoated liposomes (DPPC:DOTAP:CHL 7:1:2), and xanthohumol-loaded pectin-coated liposomes crosslinked with Ca^2+^. DSC provides critical insights into lipid bilayer phase transitions, drug–lipid interactions, and the structural modifications induced by polysaccharide coating. The thermodynamic parameters extracted from the DSC thermograms—including transition temperature (T_m_), onset temperature (T_onset_), and enthalpy change (ΔH)—collectively reveal how xanthohumol incorporation and pectin–Ca^2+^ coating alter the molecular organization and thermal stability of the liposomal bilayer.

**Figure 6:**
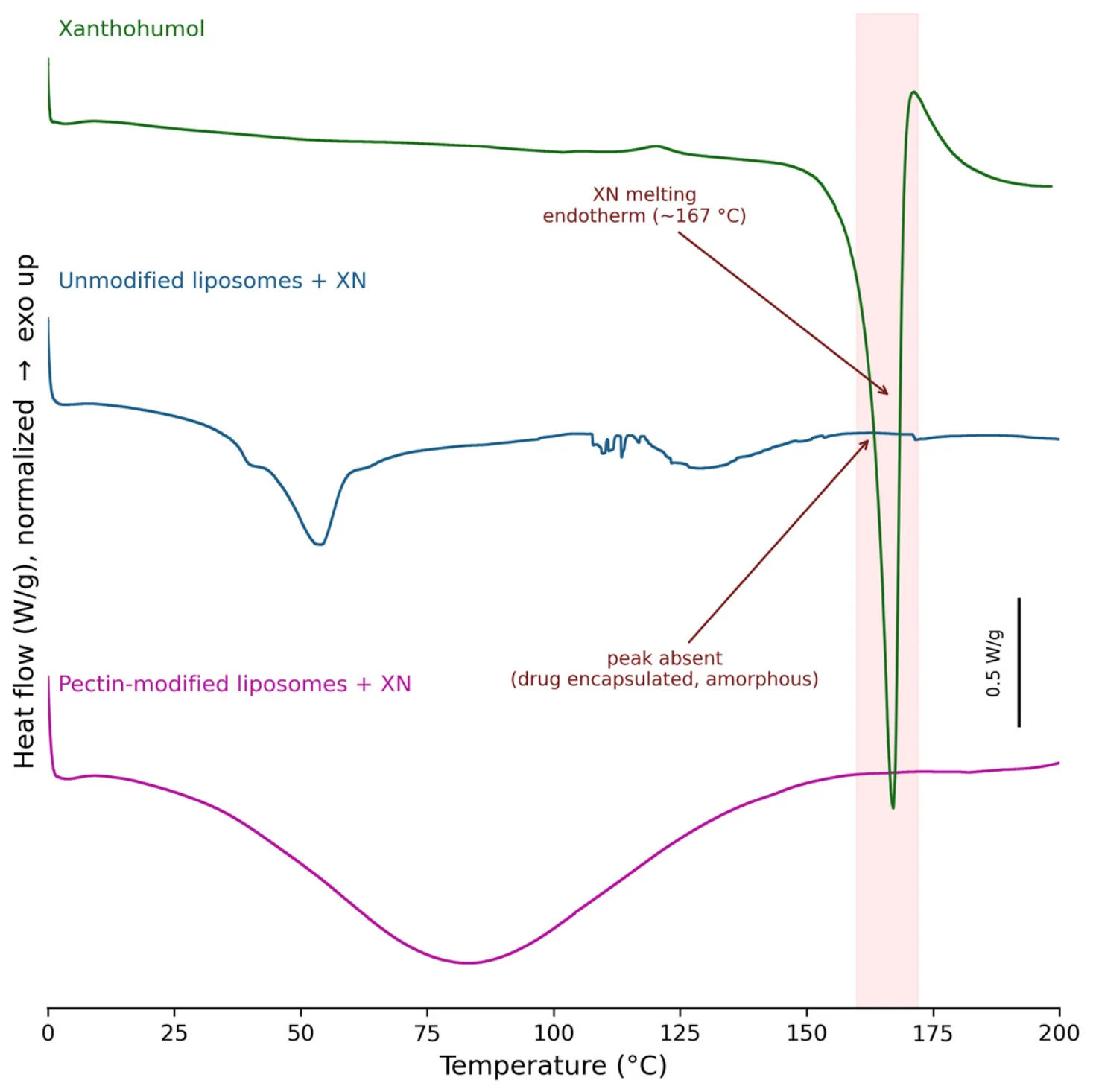
Differential scanning calorimetry thermograms of xanthohumol and the liposomal formulations. Normalized heat flow (exo up) is shown for pure xanthohumol, unmodified xanthohumol-loaded liposomes, and pectin-modified xanthohumol-loaded liposomes; curves are vertically offset for clarity.

The DSC thermogram (Figure 6) of pure xanthohumol exhibited a single, sharp endothermic peak corresponding to the melting of the crystalline drug. The peak temperature was observed at 167.13 °C with an onset at 162.07 °C and a melting enthalpy of 69.46 J/g. This melting point is consistent with literature values for xanthohumol, which typically range from 165–170 °C depending on crystalline polymorphism and heating rate [8]. The narrow transition width (~5 °C from onset to peak) indicates a well-defined, homogeneous crystalline structure with minimal impurities. No additional thermal events were observed below 160 °C, confirming the absence of polymorphic transitions or degradation processes in this temperature range and establishing the baseline thermal signature of the pure drug for comparison with the liposomal formulations.

The DSC thermogram of the uncoated xanthohumol-loaded liposomes displayed three distinct endothermic transitions, each corresponding to different structural and compositional features of the formulation. The pre-transition at 39.94 °C (onset 37.88 °C, ΔH = 0.60 J/g) corresponds to the well-documented Lβ’ → Pβ’ phase transition of DPPC, in which the tilted gel phase transforms into the ripple gel phase [45]. This transition is characteristic of fully hydrated DPPC bilayers and its preservation in the liposomal formulation indicates that the basic lamellar architecture of the phospholipid bilayer remains intact despite xanthohumol loading. However, the reduced enthalpy (0.60 J/g vs. ~1.5–2.0 J/g for pure DPPC multilamellar vesicles) and the slightly lower temperature (39.94 °C vs. ~35 °C for pure DPPC pre-transition) suggest that xanthohumol incorporation partially disrupts the cooperative chain packing in the gel phase, reducing the energetic barrier for the tilt-to-ripple transformation.

The main phase transition at 54.06 °C (onset 45.03 °C, ΔH = 19.24 J/g) represents the Pβ’ → Lα transition, in which the ripple gel phase transforms into the liquid crystalline (fluid) phase. This transition temperature is significantly elevated compared to pure DPPC, which exhibits a main transition at approximately 41 °C. The +13.1 °C upward shift in T_m_ can be attributed to two primary factors: the presence of cholesterol, which is well-known to increase the main transition temperature and broaden the transition of phospholipid bilayers by restricting acyl chain mobility and promoting lipid condensation, and the intercalation of xanthohumol within the hydrophobic core of the bilayer, which further restricts chain mobility through hydrophobic interactions and hydrogen bonding with the lipid headgroups. The reduced enthalpy is also consistent with cholesterol- and drug-induced bilayer disruption, as both cholesterol and xanthohumol create packing defects that reduce the cooperativity of the phase transition. Because the baseline thermal effects of the DPPC/cholesterol matrix are well-established [46,47], the observed structural rigidification in the surface-modified formulation can be confidently attributed to the pectin-Ca^2+^ shell.

The high-temperature endothermic event at 126.94 °C (onset 121.16 °C, ΔH = 16.03 J/g) is of particular significance as it represents a xanthohumol-related thermal signal that is distinctly different from the pure drug melting point (167.13 °C). The ~40 °C depression of this transition relative to pure xanthohumol suggests that a fraction of the encapsulated drug exists in a non-crystalline, amorphous, or molecularly dispersed state within the liposomal matrix, rather than as a separate crystalline phase. This is consistent with effective liposomal encapsulation, where the hydrophobic drug is molecularly distributed within the lipid bilayer hydrocarbon core, adopting a thermodynamically distinct state from the bulk crystalline solid [48].

The DSC thermogram of the pectin-coated, Ca^2+^-crosslinked liposomes presented a substantially different thermal profile compared to both the pure drug and the uncoated liposomal formulation. In the coated system the lipid main transition was no longer resolved as a discrete event; instead, a single broad endotherm was observed with an apparent maximum at 83.11 °C, spanning several tens of degrees. The displacement of the dominant thermal feature to markedly higher temperature, together with the loss of a distinct main transition, is consistent with a constrained, rigidified bilayer environment imposed by the crosslinked polysaccharide shell. Several interconnected mechanisms can account for this effect. First, the Ca^2+^-crosslinked pectin network at the liposome surface creates a dense, mechanically rigid hydrogel shell that physically constrains the lipid bilayer, restricting the conformational mobility of the phospholipid acyl chains and thereby elevating the energy barrier required for the gel-to-fluid phase transition(). Second, as already mentioned, the electrostatic interactions between the anionic galacturonic acid residues of pectin and the cationic trimethylammonium headgroups of DOTAP may induce localized lipid reorganization at the membrane interface, creating regions of enhanced packing density that resist thermal disruption [49].

The massive increase in the integrated endothermic heat (289.42 J/g for coated vs. 35.87 J/g total for uncoated, an ~8× increase) cannot be attributed solely to the lipid bilayer transition and strongly suggests a significant contribution from the pectin–Ca^2+^ hydrogel network itself. The dehydration of the Ca^2+^-crosslinked pectin gel during heating is an endothermic process that would contribute substantially to the overall enthalpy. As the temperature increases from 31 °C to 83 °C, the pectin–Ca^2+^ network progressively loses its structural integrity through the sequential breaking of Ca^2+^-carboxylate coordination bonds, hydrogen bond disruption, and eventual dissolution of the gel matrix—a multi-step endothermic process that is superimposed on the lipid bilayer phase transition [49]. The observed thermal effects were recorded in a single heating scan; therefore, the reported integrated heat represents the combined response of reversible (bilayer rigidification, hydrogen-bond reorganization) and irreversible (Ca^2+^-carboxylate coordination breaking, gel network destruction) contributions.

The complete absence of the pre-transition in the coated liposomes further supports the interpretation that the pectin–Ca^2+^ coating alters the phase behavior of the DPPC bilayer. The pre-transition (Lβ’ → Pβ’) requires the formation of a tilted gel phase in which the acyl chains are packed at an angle relative to the bilayer normal. The mechanical constraints imposed by the rigid pectin–Ca^2+^ shell appear to suppress this tilted packing arrangement, forcing the bilayer into a more disordered or constrained configuration that bypasses the pre-transition and transitions directly from a constrained gel-like state to the fluid phase over an extraordinarily broad temperature range.

The disappearance of the xanthohumol-related endothermic event at ~127 °C in the coated liposomes provides important evidence for improved drug encapsulation and stability in the pectin-coated formulation. In the uncoated liposomes, the 126.94 °C peak suggests the presence of a non-encapsulated or segregated drug fraction. In the coated liposomes, this peak is completely absent, indicating that (i) the pectin–Ca^2+^ shell prevents drug leakage and crystallization during sample preparation and storage, (ii) the osmotic dehydration of the liposome core during coating drives the drug more deeply into the hydrophobic bilayer core where it is molecularly dispersed, and (iii) the rigid pectin barrier prevents the release and recrystallization of xanthohumol during the DSC heating scan. This improved encapsulation stability is highly favorable for drug delivery applications, as it suggests that the pectin-coated liposomes will maintain their drug payload more effectively under physiological conditions.

From a drug delivery perspective, the elevated temperature of the composite thermal event (83.11 °C) and enhanced thermal stability of the coated liposomes suggest that the formulation will maintain its structural integrity at physiological temperatures (37 °C), remaining well below the thermal threshold and thereby preserving the gel-phase rigidity that is essential for controlled drug release. The near-neutral surface charge and thick polysaccharide shell, combined with the enhanced thermal stability, position these pectin-coated xanthohumol-loaded liposomes as promising candidates for oral or parenteral delivery applications requiring sustained release and protection from enzymatic degradation [50].

### 3.4. In Vitro Xanthohumol Release Analysis

**Figure 7:**
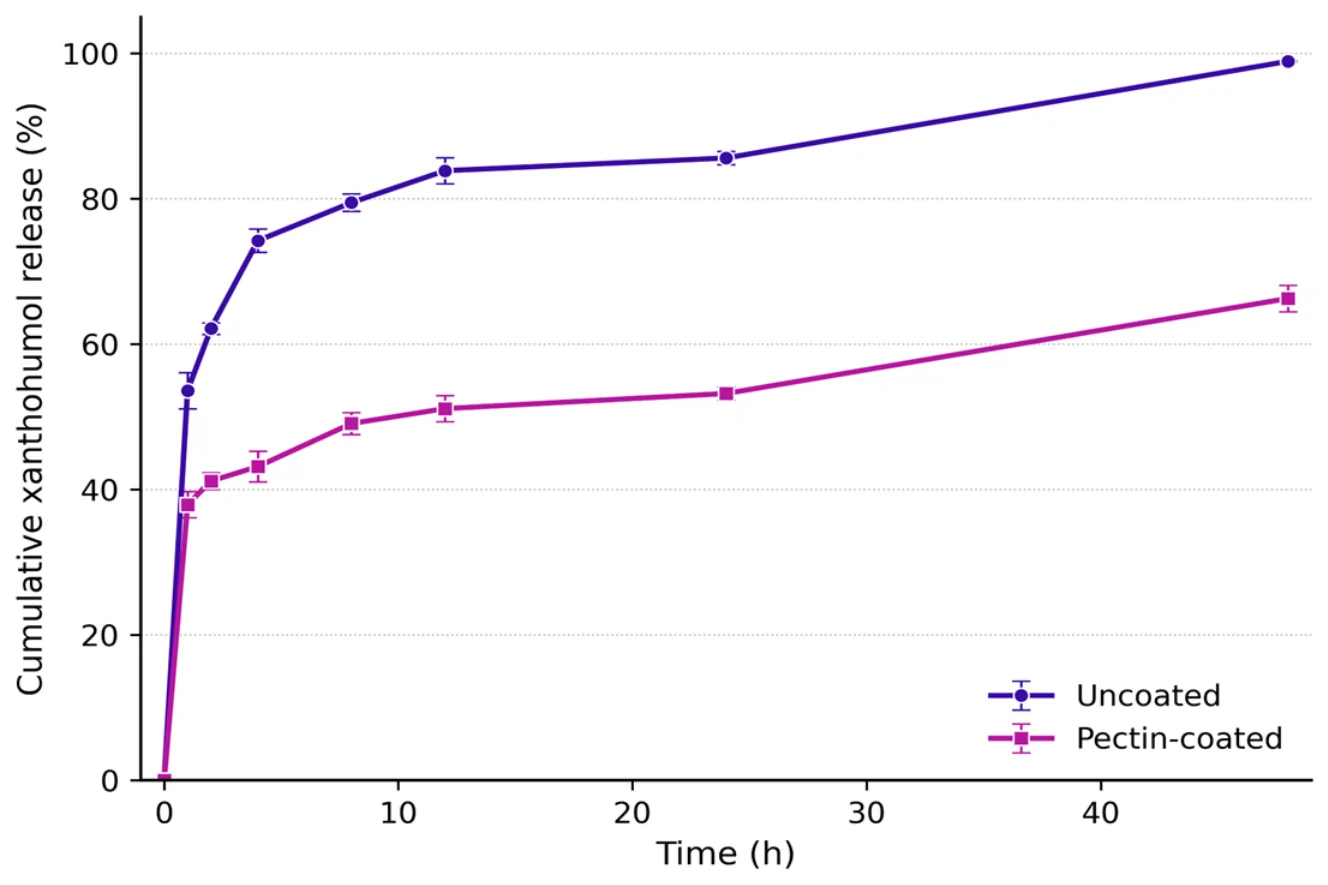
In vitro release profiles of xanthohumol from unmodified and pectin-modified liposomes.

In vitro drug release studies were conducted to evaluate the release profiles of xanthohumol from uncoated and pectin-coated liposomal formulations over a 48-h period The resulting release data was subsequently analyzed by fitting to eight established mathematical models to elucidate the underlying release mechanisms and to quantitatively assess the effect of the pectin–Ca^2+^ coating on drug release kinetics. The cumulative drug release profiles for both formulations are summarized in Figure 7.

The uncoated xanthohumol-loaded liposomes exhibited a rapid initial release, with approximately 53.60% of the encapsulated drug released within the first hour, followed by a progressively slower release phase that approached 98.93% by 48 h. This profile is characteristic of liposomal drug delivery systems, where an initial burst release is attributable to drug molecules adsorbed at or near the bilayer surface, followed by a slower diffusion-controlled release of drug molecules intercalated within the hydrophobic core of the bilayer.

The pectin-coated liposomes, by contrast, displayed an attenuated release profile. The initial burst at 1 h was reduced to 37.93%, compared to the uncoated formulation. The subsequent release continued at a substantially slower rate, reaching only 66.29% cumulative release at 48 h. These differences unambiguously demonstrate that the pectin–Ca^2+^ crosslinked shell functions as an effective diffusion barrier, retarding the release of xanthohumol from the liposomal core. The Ca^2+^-crosslinked pectin hydrogel network creates a dense, tortuous matrix that increases the effective diffusion path length and reduces the permeability of the drug through the vesicle boundary.

In vitro drug release assay for both formulations was conducted at slightly acidic conditions (pH 6.5). The data shows that the process follows a similar trend with the amount of released xanthohumol at the 48th hour determined to be 98.46% for the unmodified and 63.25% for the pectin-modified liposomes. The drug release profile of xanthohumol at pH 6.5 is presented in Figure S2.

The release data for both formulations were fitted to six mathematical models. The quality of fit for each model was evaluated by the coefficient of determination (R^2^) and summarized in Table S8.

The Peppas-Sahlin model provided the best overall fit to both formulations (R^2^ = 0.9926 for uncoated; R^2^ = 0.9888 for coated) and was therefore employed as the primary model for release mechanism identification. This model explicitly decomposes the release process into two distinct contributions, where K_1_t^m^ represents the Fickian diffusion contribution and K_2_t^2m^ represents the polymer relaxation/erosion contribution [51].

For the uncoated liposomes, the fit is dominated by the diffusional term (K_1_ = 0.66), with a negligible relaxation contribution, indicating that release is essentially diffusion-controlled. This is physically consistent with the absence of any polymeric coating on the uncoated vesicles: drug release occurs predominantly by passive diffusion of xanthohumol across the phospholipid bilayer–water interface, driven by the concentration gradient between the drug-enriched bilayer core and the surrounding release medium [11].

For the pectin-coated liposomes, both terms contribute meaningfully (K_1_ = 0.26, K_2_ = 0.10), and the diffusional fraction F = K_1_t^m^/(K_1_t^m^ + K_2_t^2m^) decreases progressively from ≈0.72 at early times to ≈0.63 by 48 h, confirming that Fickian diffusion is the primary release mechanism throughout while polymer relaxation/erosion of the pectin–Ca^2+^ shell makes a growing secondary contribution. This dual-mechanism profile is mechanistically significant and consistent with the structural characterization data: the lipid bilayer provides the primary diffusion pathway for xanthohumol, while the gradual hydration, swelling, and dissolution/erosion of the Ca^2+^-crosslinked pectin network introduces an additional rate-limiting step that complements diffusion-controlled release [52]. As the pectin shell hydrates and the Ca^2+^ crosslinks progressively dissociate, barrier permeability increases, allowing more drug to diffuse through. This combination is highly desirable for controlled delivery, uniting the predictability of diffusion-controlled release with the responsiveness of polymer erosion to environmental conditions.

The fitted release exponent (m) was found to be 0.23 for the unmodified and 0.11 for the pectin-modified liposomes, which falls below the 0.43 theoretical Fickian value for particles with spherical geometry. This indicates that the drug release is dominated by sub-Fickian restricted diffusion through the phospholipid bilayer. The coating was found to decrease the m exponent, confirming that the Ca^2+^-pectin shell provides an additional diffusional barrier.

### 3.5. In Vitro Liposomes Uptake Analysis

#### 3.5.1. Influence of the Pectin Coating on the Uptake by Phagocytic Cells

The macrophage uptake of nanoparticles is a critical determinant of their in vivo fate, as rapid clearance by the mononuclear phagocyte system (MPS) can severely limit the circulation time and targeting efficiency of drug delivery vehicles. To evaluate whether the pectin–Ca^2+^ coating confers stealth-like properties to the xanthohumol-loaded liposomes, the cellular uptake of both uncoated and pectin-coated formulations was assessed using the murine macrophage cell line RAW264.7 at concentrations of 10 and 50 µg/mL over 1 h and 2 h.

RAW264.7 macrophages were selected as a model phagocytic cell line because they express a broad spectrum of pattern recognition receptors, including scavenger receptors, complement receptors, and Fc receptors, which mediate the recognition and internalization of foreign particulates [53]. The uptake of fluorescently labelled liposomes by macrophages was quantified by measuring the relative fluorescence units (RFU) associated with the cells after washing, which provides a direct, quantitative measure of liposome internalization. Two concentrations were investigated to assess whether the stealth effect of the pectin coating is concentration dependent. At higher liposome concentrations, the surface area available for protein adsorption increases, and the difference in opsonization between coated and uncoated formulations may become more pronounced.

**Figure 8:**
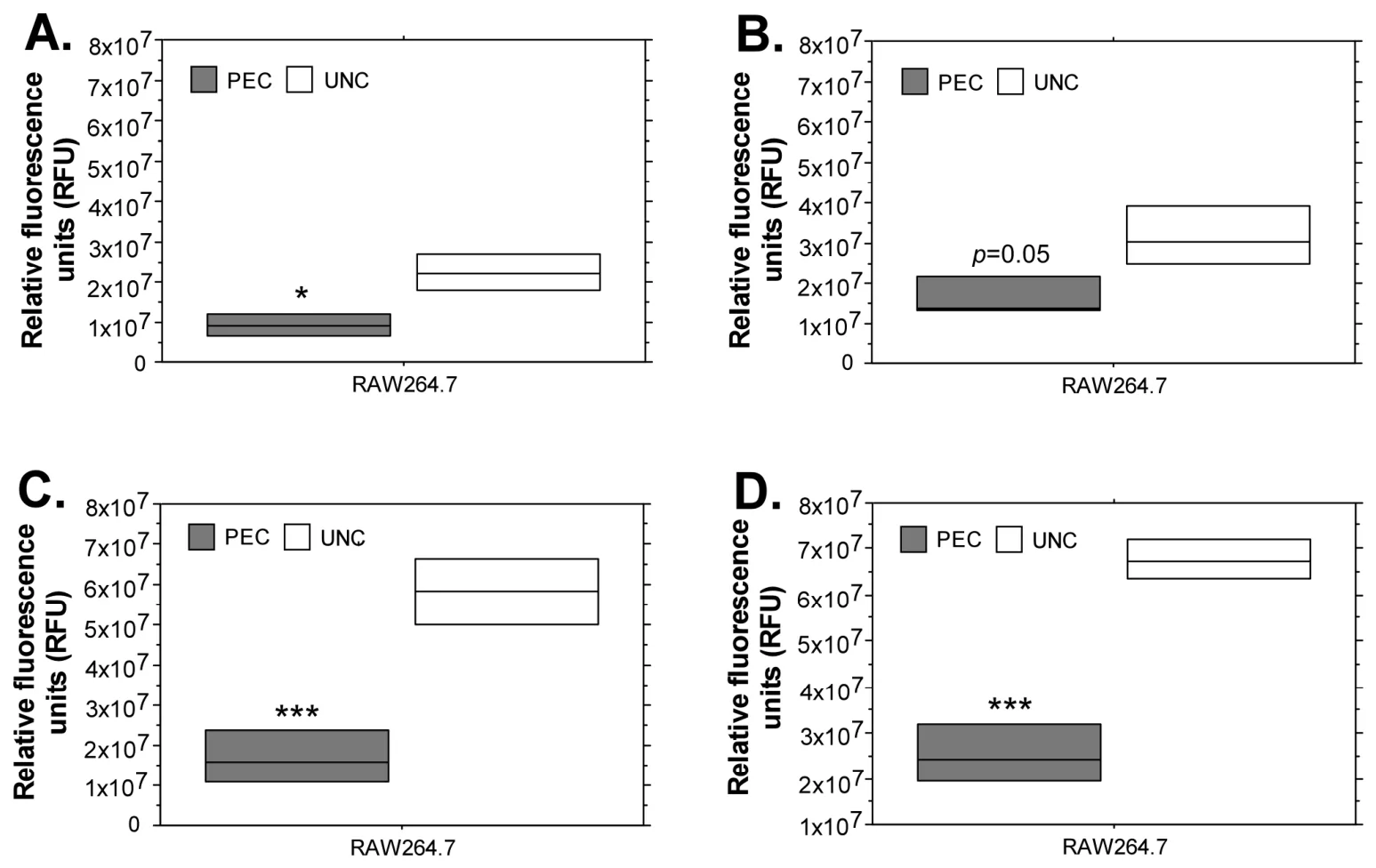
Macrophage uptake in vitro of unmodified (UNC) and pectin-modified (PEC) liposomes at a concentration of 10 µg/mL (**A**,**B**) and 50 µg/mL (**C**,**D**) for a period of 1 h (**A**,**C**) and 2 h (**B**,**D**). * *p* < 0.05; *** *p* < 0.001.

The pectin-coated liposomes exhibited a significantly lower macrophage uptake compared to the uncoated formulation at both investigated time points. At 1 h, the uptake of coated liposomes (approximately 1 × 10^7^ RFU) was 53.6% lower than that of uncoated liposomes (2.8 × 10^7^ RFU) (Figure 8A), with statistical significance at *p* < 0.05. At 2 h, the reduction was 40.0% (PEC: 2.1 × 10^7^ RFU; UNC: 3.5 × 10^7^ RFU; *p* = 0.05) (Figure 8B). At the higher concentration of 50 µg/mL, the stealth effect of the pectin coating was substantially more pronounced. At 1 h, the uptake of coated liposomes (1.6 × 10^7^ RFU) was 72.9% lower than that of uncoated liposomes (5.9 × 10^7^ RFU) (Figure 8C), with a high degree of statistical significance (*p* < 0.001). At 2 h, the reduction was 62.7% (PEC: 2.5 × 10^7^ RFU; UNC: 6.7 × 10^7^ RFU; *p* < 0.001) (Figure 8D).

The consistent and concentration-dependent reduction in macrophage uptake for the pectin-coated liposomes can be rationalized through several interconnected mechanisms that collectively create a stealth-like protective barrier around the liposomal core. The Ca^2+^-crosslinked pectin gel forms a thick, water-rich hydrogel shell, which increases the effective hydrodynamic diameter from ~160 nm to ~300 nm. This enlarged, hydrated shell reduces the efficiency of receptor-mediated binding by increasing the distance between liposome surface ligands and macrophage receptors, thereby decreasing the probability of successful recognition. At higher concentrations, the steric barrier effect is maintained because the pectin shell properties (thickness, hydration, crosslink density) are intrinsic to the formulation and do not degrade significantly over the 2-h experimental timeframe. Furthermore, the DSC data revealed that the pectin–Ca^2+^ coating increases the main transition temperature of the bilayer from 54 °C to 83 °C, indicating enhanced rigidity, which may reduce the deformability of the coated vesicles, contributing to a reduced phagocytic engulfment [54,55]. However, this mechanism is entirely hypothetical and is yet to be confirmed through specific endocytosis pathway assays. This mechanical resistance to phagocytic engulfment is independent of concentration and contributes to the baseline stealth effect observed at both 10 and 50 µg/mL.

**Figure 9:**
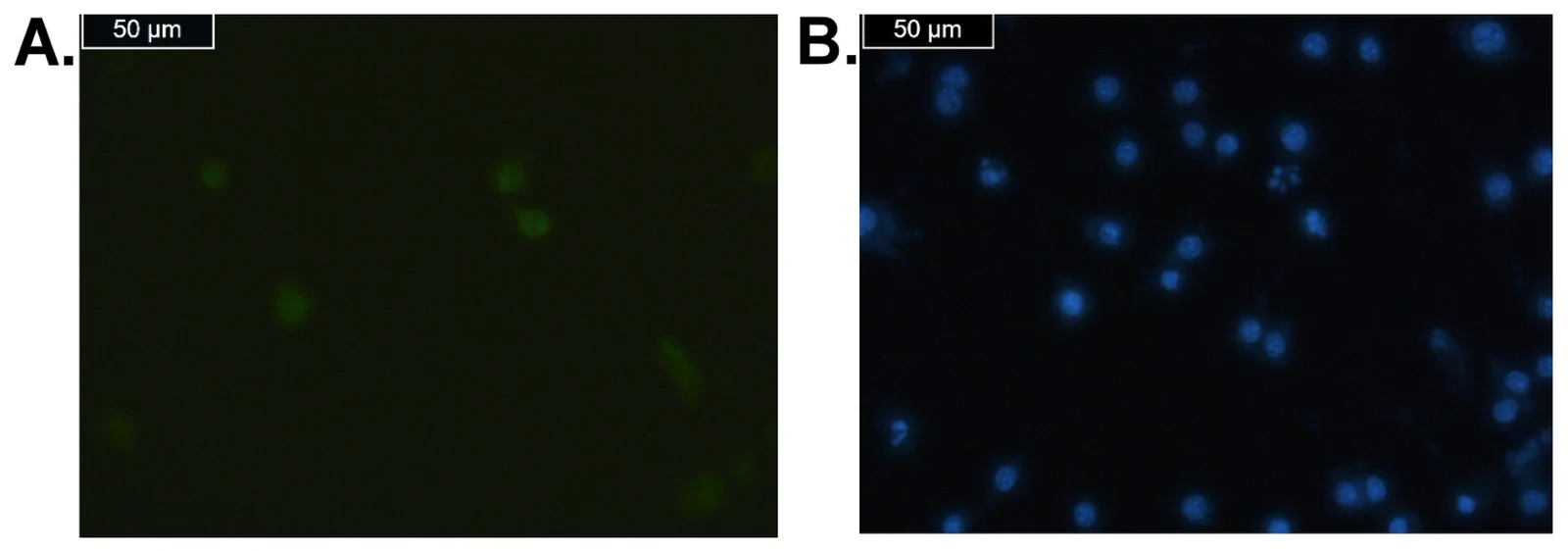
Fluorescent microscopy images of RAW264.7 cells incubated for 2 h with 50 µg/mL FITC-labelled pectin-coated liposomes: (**A**) FITC staining that indicates the macrophage uptake of pectin-modified (PEC) liposomes and (**B**) DAPI-stained nuclei of the cells in the same field of view as in image A.

The quantitative RFU data were corroborated by fluorescent microscopy imaging of RAW264.7 macrophages incubated with FITC-labelled liposomes at 50 µg/mL for 2 h (Figure 9). The FITC staining revealed that pectin-coated liposomes showed few and dim green, fluorescent signals with only sporadic punctate staining (Figure 9A). The DAPI staining confirmed significant macrophage cell densities, ruling out the possibility that the reduced FITC signal was attributable to lower cell numbers (Figure 9B). This direct visual evidence provides compelling confirmation that the pectin–Ca^2+^ coating substantially reduces the quantity of liposomes internalized by macrophages, consistent with the 63% quantitative reduction measured by fluorimetry. While pectin is often used for its mucoadhesive properties or for the preparation of colon-targeted drug delivery systems [48], the data from the conducted experiments confirms that after crosslinking with Ca^2+^, it can also function as a highly effective shield against macrophage uptake.

#### 3.5.2. Influence of the Pectin Coating on the Uptake by Non-Phagocytic Cells

To assess whether the pectin coating differentially affects uptake by phagocytic versus non-phagocytic cells, and whether any selectivity exists between cancerous and normal non-phagocytic cells, the cellular internalization for 1 h and 2 h of PEC and unmodified (UNC) liposomes both at 50 µg/mL was evaluated in two additional cell lines: A549 human lung adenocarcinoma epithelial cells (cancer, non-phagocytic) and HFFC human fetal foreskin fibroblasts (normal, non-phagocytic). These cell lines lack the specialized scavenger and complement receptors expressed by macrophages, and their nanoparticle uptake occurs primarily through clathrin-mediated endocytosis, caveolae-mediated endocytosis, and macropinocytosis rather than phagocytosis.

**Figure 10:**
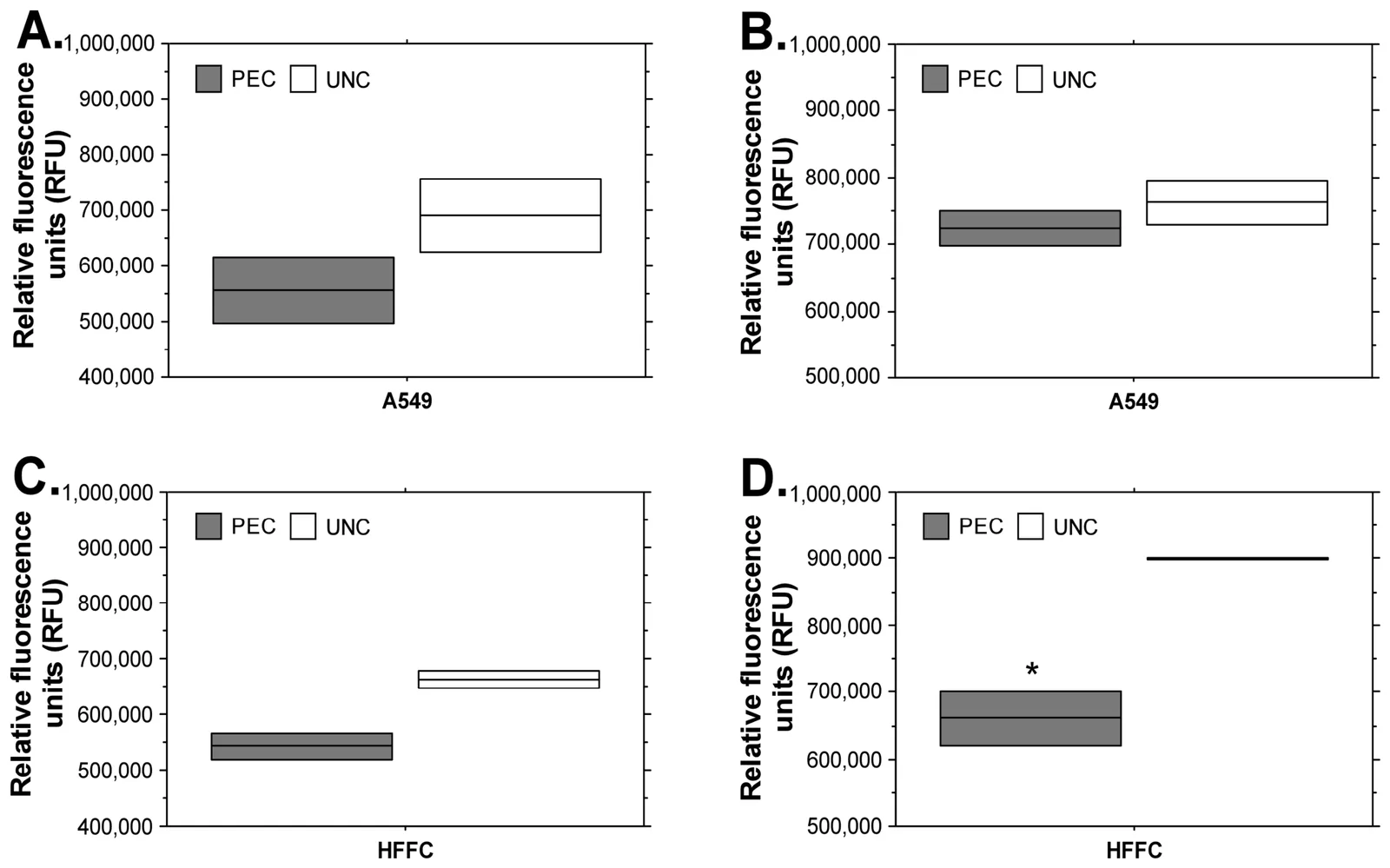
Uptake of unmodified (UNC) and pectin-modified (PEC) liposomes at a concentration of 50 µg/mL for a period of 1 h (**A**,**C**) and 2 h (**B**,**D**), by non-phagocytic cells (A549 and HFFC). * *p* < 0.05.

The A549 and HFFC data revealed that the pectin coating reduced liposome uptake by both non-phagocytic cell types, but the magnitude of this reduction was substantially smaller than that observed in macrophages. After the 1-h incubation period, the reduction was 20.3% for A549 cancer cells (PEC/UNC = 0.80) and 18.2% for HFFC normal fibroblasts (PEC/UNC = 0.82)—values that are nearly identical (Figure 10A,C). By 2 h, the A549 reduction had further diminished to only 6.5% (PEC/UNC = 0.94), indicating near-convergence of uptake between PEC and UNC in cancer cells (Figure 10B,D). In contrast, the macrophage uptake reduction remained strong at both time points (72.9% at 1 h; 62.7% at 2 h), with PEC/UNC ratios staying low (0.27 and 0.37, respectively). The resulting selectivity index (macrophage reduction/non-phagocytic cell reduction) increased dramatically from 3.6–4.0× at 1 h to 10.5× at 2 h for A549, demonstrating that the pectin coating’s stealth effect becomes progressively more selective against phagocytic cells over time.

### 3.6. In Vitro Serum Stability Study

**Figure 11:**
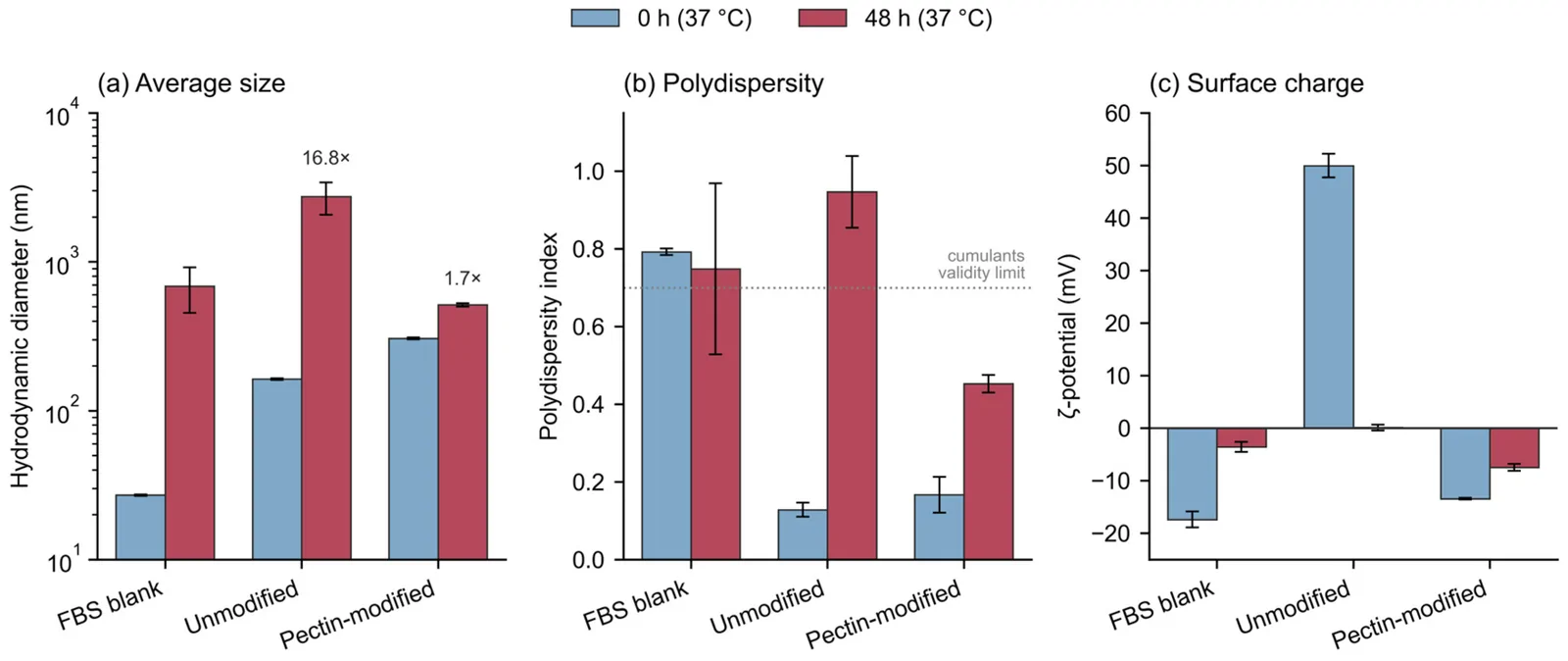
Colloidal stability of unmodified and pectin-modified liposomes before (0 h, blue) and after (48 h, red) incubation in 10% (*v*/*v*) FBS in PBS at 37 °C. (**a**) Average hydrodynamic diameter; (**b**) polydispersity index, with the dotted line marking the validity limit of the cumulants model (PDI = 0.7); (**c**) ζ-potential. Data are presented as the mean ± SD (n = 3).

To complement the uptake data, a serum stability study was conducted by incubating the unmodified and pectin-modified liposomes in 10% FBS at 37 °C. Blank FBS was kept as a reference. Prior to serum challenge, both formulations were well-defined, monodisperse colloidal dispersions (Figure 11). The unmodified cationic liposomes exhibited an average diameter of 162.9 ± 2.2 nm with a low polydispersity index (0.13) and a strongly positive ζ-potential of +47.57 ± 2.3 mV, consistent with the DOTAP-containing cationic bilayer. Deposition of the Ca^2+^-crosslinked pectin coating reversed the surface charge to −13.4 ± 0.2 mV and increased the average size to 305.4 ± 4.9 nm while maintaining a low polydispersity (0.17), confirming successful formation of the polysaccharide shell on the cationic surface without inducing heterogeneous aggregation.

The serum blank, comprising 10% (*v/v*) FBS in PBS incubated without liposomes, exhibited an apparent average size of 27.1 ± 0.3 nm at 0 h. This value should be regarded as a nominal scattering descriptor rather than a true particle size, as the accompanying polydispersity index of 0.79 lies above the accepted validity limit of the cumulants model (PDI ≤ 0.7) and reflects the intrinsically heterogeneous population of serum proteins and protein complexes.

Following 48 h of incubation, the two formulations behaved completely differently. The uncoated cationic liposomes underwent high aggregation, the average size rising from 162.9 nm to 2736 ± 669 nm, a 16.8-fold increase, accompanied by a rise in polydispersity from 0.13 to 0.95. The final size lay far above that of the serum blank at the same time point (685.7 ± 233.7 nm), establishing that the observed growth reflects genuine liposomal aggregation and not merely the scattering contribution of thermally aggregated serum proteins. In contrast, the pectin-coated liposomes remained largely intact, increasing only from 305.4 nm to 513.7 ± 14.3 nm (1.68-fold) and PDI increased from 0.17 to 0.45. The coated vesicles thus remained below the size of the serum blank itself and approximately five-fold smaller than the uncoated liposomes after the same exposure.

**Figure 12:**
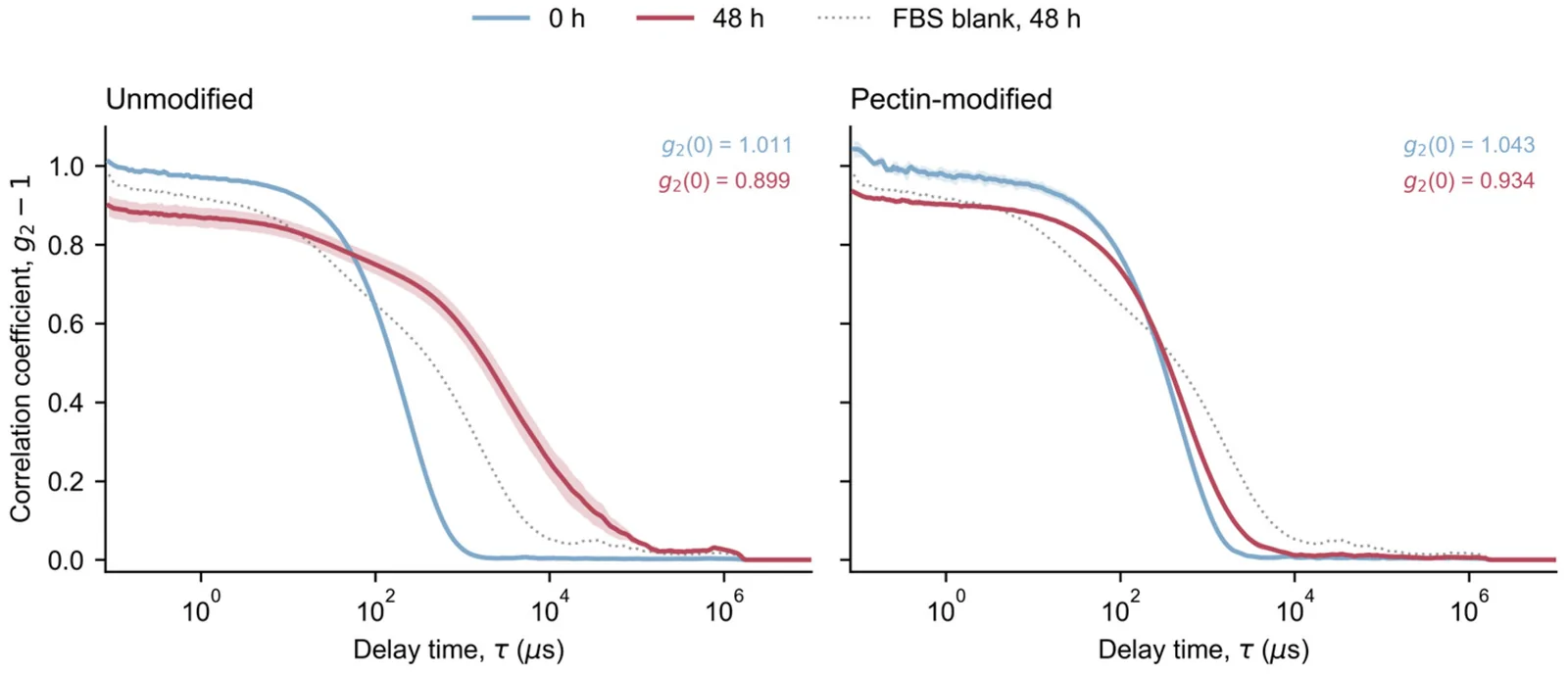
Intensity autocorrelation functions of unmodified and pectin-modified liposomes before (0 h, blue) and after (48 h, red) incubation in 10% (*v*/*v*) FBS in PBS at 37 °C.

The raw intensity autocorrelation functions were examined directly as a model-free corroboration of the size data (Figure 12). Two features of the correlogram are informative: the intercept, g_2_(0), which reports the quality of the scattering signal and falls in the presence of large, slowly sedimenting aggregates; and the characteristic decay time, which is proportional to the hydrodynamic size of the scattering population and is obtained without any fitting model.

Prior to serum exposure, both formulations produced smooth, monomodal correlograms with intercepts close to unity (1.011 for the uncoated and 1.043 for the pectin-coated liposomes) and half-decay times, τ_½_, of 160 µs and 294 µs, respectively, ordering as their hydrodynamic diameters did. After 48 h in serum, the uncoated formulation exhibited a depressed intercept (0.899) together with a markedly retarded, multimodal decay, τ_½_ lengthening 16.6-fold from 160 to 2662 µs—both hallmarks of the emergence of large, slowly diffusing aggregate populations. The pectin-coated formulation, by contrast, retained a smooth, monomodal correlogram closely resembling its initial profile, with τ_½_ lengthening only modestly from 294 to 410 µs (1.39-fold) and remaining faster-decaying than the serum blank itself (512 µs).

The evolution of the ζ-potential provides a mechanistic account of the size behaviour. The strongly cationic liposomes lost essentially all of their surface charge upon serum exposure, the ζ-potential falling from +47.57 ± 2.3 mV to +0.1 ± 0.6 mV, a value statistically indistinguishable from zero. This complete neutralisation is the direct signature of the adsorption of predominantly anionic serum proteins onto the positively charged bilayer, which removes the electrostatic repulsion responsible for colloidal stability and thereby precipitates the massive aggregation observed. The pectin-coated formulation, by contrast, retained a substantially greater fraction of its initial charge (−7.5 ± 0.7 mV, corresponding to 56% of its initial magnitude), consistent with a partially preserved surface layer and a more limited interaction with serum proteins.

The behaviour of the unmodified cationic liposomes illustrates the fundamental liability that the coating is intended to address. The pronounced positive surface charge that favours interaction with cell membranes also promotes rapid adsorption of the predominantly anionic serum proteins. The observed collapse of the ζ-potential to near-neutrality, together with the 16.8-fold increase in hydrodynamic diameter and the appearance of a slow, multimodal decay in the autocorrelation function, is consistent with protein corona formation, loss of electrostatic stabilisation and subsequent uncontrolled aggregation [56]. Cationic liposomes in their unmodified state are therefore poorly suited to any application requiring prolonged residence in a protein-rich biological milieu.

The protection afforded by the pectin coating is most readily rationalised in terms of the coherence of the Ca^2+^-crosslinked network rather than the magnitude of the surface potential it generates. This distinction is important, and is made clear by the magnitude of the zeta potential itself: the pectin-coated vesicles carry an initial surface charge of only −13.4 mV, a value well below the ±20–30 mV threshold conventionally associated with electrostatically stabilised colloids, and one which falls further to −7.5 mV after serum exposure. On a purely electrostatic account, such a formulation would be expected to be colloidally fragile; in fact it proved markedly resistant to aggregation, its hydrodynamic diameter increasing by less than 70% over 48 h at 37 °C in the presence of serum. Electrostatic repulsion is thus not the operative stabilising mechanism, and the protection may instead be attributed to the steric and mechanical barrier presented by the crosslinked polysaccharide shell.

### 3.7. In Vitro Antitumor Activity

**Figure 13:**
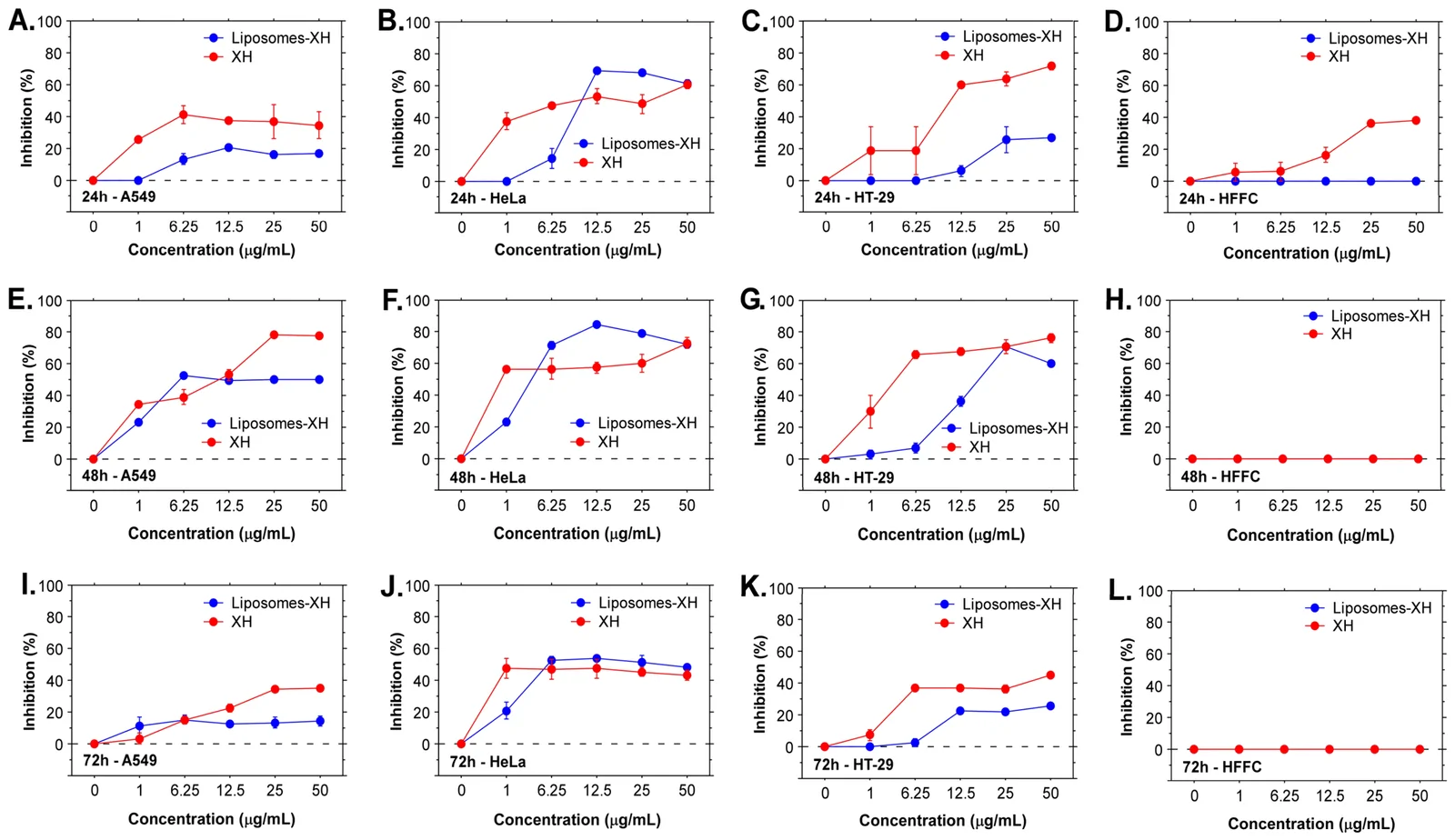
Cytotoxicity of xanthohumol and xanthohumol-loaded liposomes against human tumor and normal cell lines. Graphs (**A**,**E**,**I**) show the percentage inhibition of A549 cells metabolic activity measured at three different time points (24, 48, and 72 h); charts (**B**,**F**,**J**) show results for HeLa cells; graphs (**C**,**G**,**K**) illustrate data for HT-29 cell line, and (**D**,**H**,**L**) show the results for normal HFFC fibroblasts. Data are presented as ± standard error of the mean (±SEM) and compared with the effect of the pure substance at the same concentration.

**Figure 14:**
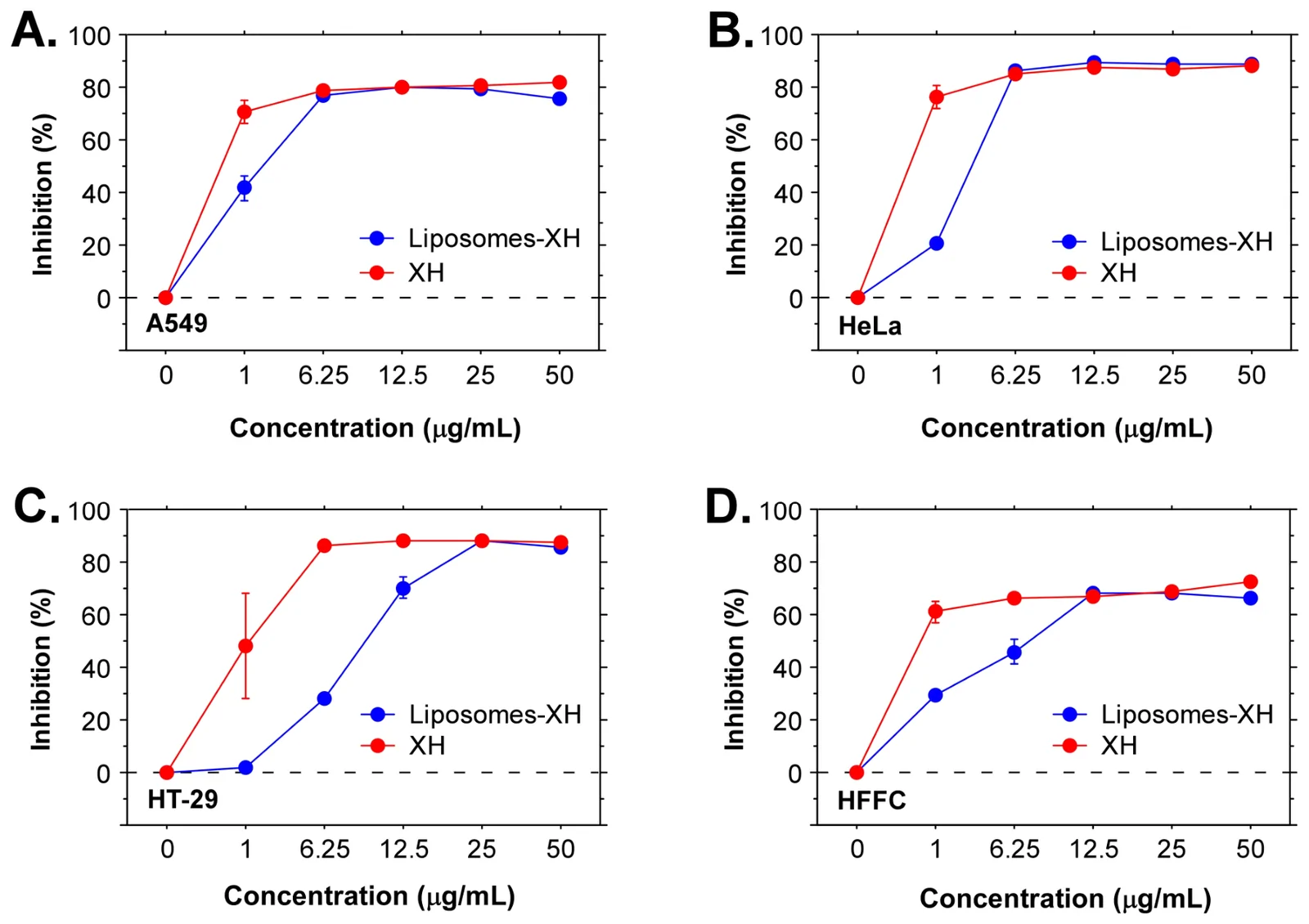
Inhibitory effects on A549 (**A**), HeLa (**B**), HT-29 (**C**) and HFFC (**D**) cell viability measured by NR assay after 48 h treatment with XH or XH-loaded liposomes. Data are presented as ± standard error of the mean (±SEM) and compared with the effect of the pure substance at the same concentration.

To evaluate whether the pectin-coated xanthohumol-loaded liposomes retain the antitumor activity of the free drug, and to assess the impact of liposomal encapsulation and sustained release on cytotoxic potency, IC_50_ values were determined for both free xanthohumol (XH) and pectin-coated XH-loaded liposomes (Liposomes-XH) across a panel of human tumor cell lines (A549 lung adenocarcinoma, HeLa cervical carcinoma, HT-29 colorectal adenocarcinoma) and normal human fibroblasts (HFFC) using two complementary in vitro assays: the MTT assay and the Neutral Red assay (Figure 13 and Figure 14).

The MTT assay measures mitochondrial dehydrogenase activity as a proxy for metabolic viability, reflecting the energetic status of the cell, its viability and metabolic activity. The Neutral Red assay quantifies the accumulation of the dye in lysosomes, providing a measure of lysosomal membrane integrity and general cell health. These two assays are complementary because they target distinct cellular compartments (mitochondria vs. lysosomes) and can respond differently to drug-induced stress depending on the mechanism of action. Xanthohumol is known to exert its cytotoxic effects through multiple pathways, including mitochondrial dysfunction, reactive oxygen species generation, and cell cycle arrest, making the dual-assay approach particularly appropriate for evaluation of its cytotoxic profile.

To complement the single-concentration IC_50_ determinations and to elucidate the time-dependent cytotoxicity profile of free versus liposomal xanthohumol, a comprehensive dose–response analysis was performed using the MTT assay at three time points (24, 48, and 72 h) across a concentration range of 0–50 µg/mL (Figure 13). The dose–response curves revealed a striking and informative temporal evolution in the relative cytotoxic potency of the two formulations, reflecting the interplay between free drug availability and sustained-release kinetics. At 24 h, free xanthohumol exhibited a steep dose–response curve with inhibition rising from 0% at 0 µg/mL to a peak of ~43% at 6.25 µg/mL, followed by a slight decline at higher concentrations (38–36% at 12.5–50 µg/mL). This peak-and-plateau pattern is characteristic of drugs that exert their cytotoxic effect rapidly and then encounter cellular resistance mechanisms or metabolic adaptation at sustained high concentrations. The liposomal formulation, by contrast, showed a markedly attenuated response at 24 h, with inhibition reaching only ~21% at 12.5 µg/mL before plateauing at ~17%. This dramatic difference is fully consistent with the sustained-release profile: at 24 h, the liposomes have released only a fraction of their encapsulated xanthohumol payload (t_50_ ~11 h from Section 3.4), resulting in substantially lower effective drug concentrations at the cellular level compared to the immediately bioavailable free drug. At 48 h, the gap between the two formulations narrowed substantially reflecting the progressive release of xanthohumol from the liposomal matrix. Free xanthohumol achieved strong inhibition across the concentration range, peaking at ~77% at 25–50 µg/mL. The liposomal formulation showed a more gradual dose–response, reaching ~50% inhibition at 12.5 µg/mL and maintaining this level through 50 µg/mL. Notably, at 12.5 µg/mL, both formulations achieved comparable inhibition (~50%) of A549 cells, representing a convergence point where the sustained release from liposomes has delivered sufficient cumulative drug to match the immediate exposure of free xanthohumol. This convergence is therapeutically significant because it demonstrates that the liposomal formulation can achieve equivalent cytotoxic effects at moderate concentrations given sufficient time for the sustained-release mechanism to operate.

HeLa cells showed the highest sensitivity for xanthohumol-loaded liposomes among all tested cell lines—notable inhibition of cellular methabolic activity after treatment with higher than 6.25 µg/mL sample concentrations (Figure 13B,F,J).

The Neutral Red assay provided a consistent and interpretable pattern across all four cell lines (Figure 14), with the liposomal formulation exhibiting higher IC_50_ values than free xanthohumol (Table 2). The elevation of IC_50_ for the liposomal formulation across all cell lines in the NR assay is fully consistent with the sustained-release profile established in the drug release studies. The pectin-coated liposomes exhibit a release half-life of approximately 11 h (t_50_), meaning that at the 48-h timepoint of the cytotoxicity assay, only a fraction of the encapsulated xanthohumol has been released from the liposomal matrix. The free drug, by contrast, is immediately bioavailable and can exert its full cytotoxic effect from the moment of administration. Consequently, the effective drug concentration experienced by cells treated with the liposomal formulation is lower than that experienced by cells treated with an equivalent nominal concentration of free drug, resulting in the observed higher IC_50_ values for liposome-treated cells (Table 2).

This result indicates that the pectin-coated liposomes effectively deliver xanthohumol to cells, despite the sustained-release barrier posed by the pectin–Ca^2+^ shell. Several mechanisms may account for this drug delivery property. First, the liposomal formulation may exploit the enhanced endocytic activity of tumor cells, which overexpress clathrin-coated pit machinery and exhibit elevated macropinocytic rates characteristic of the transformed phenotype [57]; however, this hypothesis is yet to be confirmed. The liposomes, with their ~300 nm hydrodynamic diameter (DLS data), fall within the reported size range, which supports endocytic internalization of the vesicles, delivering a concentrated payload of xanthohumol into the cell [58]. Free xanthohumol, by contrast, relies on passive diffusion across the plasma membrane—a process that may be limited by its poor aqueous solubility and rapid efflux by multidrug resistance pumps [59,60].

The inclusion of normal HFFC fibroblasts in the assay panel enables a direct assessment of antitumor activity through the selectivity index (SI), defined as the ratio of the IC_50_ for normal cells to that in the corresponding tumor cell line; higher SI values indicate greater selectivity for malignant cells and a more favorable safety profile. For free xanthohumol, the SI values were close to unity across the panel (A549, 1.08; HeLa, 1.10; HT-29, 0.75), indicating essentially comparable toxicity toward malignant and normal cells. Encapsulation markedly improved this profile in two of the three cancer lines: the SI rose to 3.35 for A549 (a 3.1-fold increase) and to 2.12 for HeLa (a 1.9-fold increase), whereas HT-29 remained essentially unchanged and below unity (0.74 vs. 0.75), indicating no selectivity gain in this line.

The improvement observed in higher SI values for A549 and HeLa cells is consistent with the sustained-release kinetics of the liposomal formulation, which establish a more favorable exposure profile for cancer cells—these typically exhibit higher metabolic activity, increased endocytic rates, and greater sensitivity to prolonged low-dose drug exposure than normal fibroblasts. HFFC cells, with their lower endocytic activity and more robust homeostatic mechanisms, appear better able to tolerate the slower, sustained delivery from the liposomes than the immediate high-concentration pulse produced by the free drug [61]. The absence of any selectivity gain in HT-29 cells indicates that this benefit is cell-line dependent and is not a general property of the formulation.

## 4. Conclusions

In the present study, pectin-coated, Ca^2+^-crosslinked cationic liposomes were successfully developed and optimized as a multifunctional delivery platform for hydrophobic anticancer agents using xanthohumol as a model compound. Surface modification through electrostatic deposition followed by Ca^2+^-mediated crosslinking produced a stable polymeric shell with essentially complete retention of the pre-encapsulated drug and favorable physicochemical characteristics. The optimized coating significantly reduced the initial burst release and provided sustained drug release over 48 h, while simultaneously improving serum stability and reducing macrophage uptake. Importantly, these beneficial effects were achieved, while improving cancer-cell selectivity index for A549 and HeLa cells relative to normal fibroblasts.

Taken together, the results demonstrate that pectin surface functionalization effectively addresses several major limitations of conventional cationic liposomes, including poor release control, limited stability under physiological conditions, and rapid recognition by the mononuclear phagocyte system. Rather than improving a single formulation parameter, the developed coating strategy simultaneously enhances multiple properties that are critical for systemic drug delivery. These findings highlight the potential of pectin-coated liposomes as a versatile platform for the delivery of hydrophobic anticancer agents. Despite that, however, this study features a couple of limitations. Firstly, the drug release was assessed only in buffer media (pH 7.4 and 6.5), while release in serum-containing medium was not quantified and is identified as an objective for future work. The stealth-like properties, colloidal stability and cancer-cell selectivity were evaluated solely on in vitro models, which cannot fully replicate the complex conditions of the in vivo models. Additionally, while the baseline sustained-release mechanism has been established, the exact intracellular trafficking and release pathways following endocytosis remain to be thoroughly established. Future studies will focus on pharmacokinetic, biodistribution, and in vivo therapeutic investigations to further evaluate the translational potential of the developed delivery system.

## Figures and Tables

**Table 1:** L9 Taguchi design matrix with the formulation variables at a fixed pectin:liposome ratio of 3:1 (*w*/*w*) and corresponding pectin (PEC)-modified models (n = 3).

Model	Pectin Concentration		CaCl_2_ Concentration		Incubation Time		Average Size ± SD (nm)	Ζ-Potential ± SD (mV)	PDI ± SD
	Level	mg/mL	Level	mM	Level	min			
PEC1	1	0.5	1	1	1	30	274.6 ± 5.6	−30.12 ± 0.33	0.25 ± 0.01
PEC2	1	0.5	2	5	2	60	305.4 ± 4.0	−13.43 ± 0.13	0.17 ± 0.04
PEC3	1	0.5	3	10	3	120	300.6 ± 2.3	−10.79 ± 0.06	0.18 ± 0.04
PEC4	2	1.0	1	1	2	60	272.3 ± 3.2	−26.48 ± 0.37	0.15 ± 0.01
PEC5	2	1.0	2	5	3	120	294.1 ± 1.4	−13.97 ± 0.18	0.20 ± 0.02
PEC6	2	1.0	3	10	1	30	361.9 ± 9.4	−10.94 ± 0.43	0.28 ± 0.01
PEC7	3	2.0	1	1	3	120	362.5 ± 2.1	−22.69 ± 0.77	0.20 ± 0.01
PEC8	3	2.0	2	5	1	30	484.9 ± 4.2	−14.19 ± 0.54	0.29 ± 0.01
PEC9	3	2.0	3	10	2	60	454.0 ± 6.7	−11.62 ± 0.02	0.29 ± 0.01

**Table 2:** Mean IC_50_ (µg/mL) and selectivity index (SI) values determined by NR assay following 48-h treatment with xanthohumol (XH) and liposomes loaded with XH.

Cell Lines	IC_50_ Liposomes-XH	SI Liposomes-XH	IC_50_ XH	SI XH
A549	2.11	3.35	1.69	1.08
HeLa	3.34	2.12	1.66	1.1
HT-29	9.56	0.74	2.45	0.75
HFFC	7.07	-	1.83	-

## Data Availability

The raw data supporting the conclusions of this article will be made available by the authors on request.

## Supplementary Materials

The following supporting information can be downloaded at: https://www.mdpi.com/article/10.3390/pharmaceutics18101275/s1, Table S1: Response table for Signal-to-Noise ratios for the average size model. Table S2: Statistical data for the effect of pectin coating parameters on the average particle size. Table S3: Response table for Signal-to-Noise ratios for the ζ-potential model. Table S4: Statistical data for the effect of pectin coating parameters on the ζ-potential. Table S5: Response table for Signal-to-Noise ratios for the polydispersity index model. Table S6: Statistical for the effect of pectin coating parameters on the PDI. Table S7: Thermodynamic parameters of pectin binding to xanthohumol-loaded cationic liposomes determined by isothermal titration calorimetry at 25 °C. Table S8: R2—values of the fitted mathematical models for xanthohumol release profile. Figure S1: IFT fit of SAXS scattering data and pair distance distribution functions p(r) for uncoated (A,B) and pectin-coated (C,D) liposomes. Figure S2: In vitro release profiles of xanthohumol from unmodified and pectin-modified liposomes at pH 6.5.

## Abbreviations

The following abbreviations are used in this manuscript:

DMEM	Dulbecco’s Modified Eagle Medium
DOTAP	1,2-Dioleoyloxy-3-trimethylammonium-propane chloride
DPPC	1,2-Dipalmitoyl-sn-glycero-3-phosphocholine
DLS	Dynamic light scattering
DSC	Differential scanning calorimetry
EE	Entrapment efficiency
FBS	Fetal bovine serum
FITC	Fluorescein isothiocyanate
HFFC	Human foreskin fibroblasts
HPLC	High-performance liquid chromatography
HSD	Honestly significant difference
ITC	Isothermal titration calorimetry
MPS	Mononuclear phagocyte system
MTT	3-(4,5-dimethylthiazol-2-yl)-2,5-diphenyltetrazolium bromide
NR	Neutral red
PBS	Phosphate-buffered saline
PDI	Polydispersity index
R^2^	Coefficient of determination
SAXS	Small-angle X-ray scattering
S/N	Signal-to-noise ratio
TEM	Transmission electron microscopy

## References

[1] Amin A.R.M.R., Kucuk O., Khuri F.R., Shin D.M. (2009). Perspectives for Cancer Prevention with Natural Compounds. J. Clin. Oncol..

[2] Warias P., Plewa P., Poniewierska-Baran A. (2024). Resveratrol, Piceatannol, Curcumin, and Quercetin as Therapeutic Targets in Gastric Cancer—Mechanisms and Clinical Implications for Natural Products. Molecules.

[3] Kopustinskiene D.M., Jakstas V., Savickas A., Bernatoniene J. (2020). Flavonoids as Anticancer Agents. Nutrients.

[4] Rabbani S.A., Sharma S., El-Tanani M., Khurana S., Saini M., Yadav M., Kumar R., El-Tanani Y. (2026). Plant-Derived Polyphenols in Cancer Therapy: Bridging Molecular Mechanisms and Bioavailability Toward Clinical Translation. Pharmaceutics.

[5] Jia W., Zhou L., Li L., Zhou P., Shen Z. (2023). Nano-Based Drug Delivery of Polyphenolic Compounds for Cancer Treatment: Progress, Opportunities, and Challenges. Pharmaceuticals.

[6] Radeva L., Yordanov Y., Spassova I., Kovacheva D., Tibi I.P.-E., Zaharieva M.M., Kaleva M., Najdenski H., Petrov P.D., Tzankova V. (2024). Incorporation of Resveratrol-Hydroxypropyl-β-Cyclodextrin Complexes into Hydrogel Formulation for Wound Treatment. Gels.

[7] Jiang C.-H., Sun T.-L., Xiang D.-X., Wei S.-S., Li W.-Q. (2018). Anticancer Activity and Mechanism of Xanthohumol: A Prenylated Flavonoid From Hops (*Humulus Lupulus* L.). Front. Pharmacol..

[8] Harish V., Tewari D., Mohd S., Govindaiah P., Babu M.R., Kumar R., Gulati M., Gowthamarajan K., Madhunapantula S.V., Chellappan D.K. (2022). Quality by Design Based Formulation of Xanthohumol Loaded Solid Lipid Nanoparticles with Improved Bioavailability and Anticancer Effect against PC-3 Cells. Pharmaceutics.

[9] Zahariev N., Penkov D., Boyuklieva R., Simeonov P., Lukova P., Ardasheva R., Katsarov P. (2026). Biopolymer Casein–Pullulan Coating of Fe_3_O_4_ Nanocomposites for Xanthohumol Encapsulation and Delivery. Polymers.

[10] Inglut C.T., Sorrin A.J., Kuruppu T., Vig S., Cicalo J., Ahmad H., Huang H.-C. (2020). Immunological and Toxicological Considerations for the Design of Liposomes. Nanomaterials.

[11] Lee Y., Thompson D.H. (2017). Stimuli-responsive Liposomes for Drug Delivery. WIREs Nanomed. Nanobiotechnol..

[12] Cao Y., Dong X., Chen X. (2022). Polymer-Modified Liposomes for Drug Delivery: From Fundamentals to Applications. Pharmaceutics.

[13] Jelezova I., Drakalska E., Momekova D., Shalimova N., Momekov G., Konstantinov S., Rangelov S., Pispas S. (2015). Curcumin Loaded pH-Sensitive Hybrid Lipid/Block Copolymer Nanosized Drug Delivery Systems. Eur. J. Pharm. Sci..

[14] Zhuang J., Ping Q., Song Y., Qi J., Cui Z. (2010). Effects of Chitosan Coating on Physical Properties and Pharmacokinetic Behavior of Mitoxantrone Liposomes. Int. J. Nanomed..

[15] Wang J., Viola M., Migliorini C., Paoletti L., Arpicco S., Di Meo C., Matricardi P. (2023). Polysaccharide-Based Nanogels to Overcome Mucus, Skin, Cornea, and Blood-Brain Barriers: A Review. Pharmaceutics.

[16] Liu K., Jiang X., Hunziker P. (2016). Carbohydrate-Based Amphiphilic Nano Delivery Systems for Cancer Therapy. Nanoscale.

[17] Minzanova S., Mironov V., Arkhipova D., Khabibullina A., Mironova L., Zakirova Y., Milyukov V. (2018). Biological Activity and Pharmacological Application of Pectic Polysaccharides: A Review. Polymers.

[18] Hari K.D., Garcia C.V., Shin G.-H., Kim J.-T. (2021). Improvement of the UV Barrier and Antibacterial Properties of Crosslinked Pectin/Zinc Oxide Bionanocomposite Films. Polymers.

[19] Simeonov P., Todorova V., Batsalova T., Dzhambazov B., Ivanova S., Katsarov P. (2026). Biopolymer Surface Modification as a Strategy for Conferring “Stealth-like” Characteristics of Xanthohumol-Loaded Liposomes. Polymers.

[20] Wang H., Gou R., Li W., Chen Z., Gu C., Shi S., Zou L., Li H. (2025). Targeting Delivery of Dexamethasone to Inflamed Joints by Albumin-Binding Peptide Modified Liposomes for Rheumatoid Arthritis Therapy. Int. J. Nanomed..

[21] Arunachalam K., Sreeja P.S. (2025). MTT Assay Protocol. Advanced Cell and Molecular Techniques.

[22] Repetto G., Del Peso A., Zurita J.L. (2008). Neutral Red Uptake Assay for the Estimation of Cell Viability/Cytotoxicity. Nat. Protoc..

[23] Batsalova T., Vasil’kov A., Moten D., Voronova A., Teneva I., Naumkin A., Dzhambazov B. (2023). Bimetallic Gold–Iron Oxide Nanoparticles as Carriers of Methotrexate: Perspective Tools for Biomedical Applications. Appl. Sci..

[24] Correa S., Boehnke N., Deiss-Yehiely E., Hammond P.T. (2019). Solution Conditions Tune and Optimize Loading of Therapeutic Polyelectrolytes into Layer-by-Layer Functionalized Liposomes. ACS Nano.

[25] Obomighie I., Prentice I.J., Lewin-Jones P., Bachtiger F., Ramsay N., Kishi-Itakura C., Goldberg M.W., Hawkins T.J., Sprittles J.E., Knight H. (2025). Understanding Pectin Cross-Linking in Plant Cell Walls. Commun. Biol..

[26] O’Shaughnessy B., Vavylonis D. (2003). Irreversible Adsorption from Dilute Polymer Solutions. Eur. Phys. J. E.

[27] Doan T.H.Y., Adachi Y. (2020). Relaxation of Adsorbed Layer Thickness and Electrophoresis of Polystyrene Latex Particles after Overshooting of Polyelectrolytes with Different Charge Density. Colloids Surf. A Physicochem. Eng. Asp..

[28] Yu C., Guan J., Chen K., Bae S.C., Granick S. (2013). Single-Molecule Observation of Long Jumps in Polymer Adsorption. ACS Nano.

[29] Gawkowska D., Cybulska J., Zdunek A. (2018). Structure-Related Gelling of Pectins and Linking with Other Natural Compounds: A Review. Polymers.

[30] Gangurde P., Mahmoudzadeh M., Gounani Z., Koivuniemi A., Laurén P., Lajunen T., Laaksonen T. (2023). Development of Robust Cationic Light-Activated Thermosensitive Liposomes: Choosing the Right Lipids. Mol. Pharm..

[31] Wurm F., Rietzler B., Pham T., Bechtold T. (2020). Multivalent Ions as Reactive Crosslinkers for Biopolymers—A Review. Molecules.

[32] Guo C., Gemeinhart R.A. (2008). Understanding the Adsorption Mechanism of Chitosan onto Poly(Lactide-Co-Glycolide) Particles. Eur. J. Pharm. Biopharm..

[33] Pelssers E.G.M., Stuart M.A.C., Fleer G.J. (1990). Kinetics of Bridging Flocculation. Role of Relaxations in the Polymer Layer. Faraday Trans..

[34] Ruan Z., Wu A., Bürger R., Betancourt F., Ordoñez R., Wang J., Wang S., Wang Y. (2021). A Population Balance Model for Shear-Induced Polymer-Bridging Flocculation of Total Tailings. Minerals.

[35] Pasarin D., Ghizdareanu A.-I., Enascuta C.E., Matei C.B., Bilbie C., Paraschiv-Palada L., Veres P.-A. (2023). Coating Materials to Increase the Stability of Liposomes. Polymers.

[36] Danaei M., Dehghankhold M., Ataei S., Hasanzadeh Davarani F., Javanmard R., Dokhani A., Khorasani S., Mozafari M.R. (2018). Impact of Particle Size and Polydispersity Index on the Clinical Applications of Lipidic Nanocarrier Systems. Pharmaceutics.

[37] Mihailescu M., Worcester D.L., Carroll C.L., Chamberlin A.R., White S.H. (2023). DOTAP: Structure, Hydration, and the Counterion Effect. Biophys. J..

[38] Lutta A., Liu Q., Pedersen G.K., Dong M., Grohganz H., Nielsen L.H., Schmidt S.T. (2025). Microfluidic Fabrication of Pectin-Coated Liposomes for Drug Delivery. Drug Deliv. Transl. Res..

[39] Mertins O., Dimova R. (2011). Binding of Chitosan to Phospholipid Vesicles Studied with Isothermal Titration Calorimetry. Langmuir.

[40] Camilleri D., Attard K., Lia F. (2025). Formulation and Evaluation of Liposome-Encapsulated Phenolic Compounds from Olive Mill Waste: Insights into Encapsulation Efficiency, Antioxidant, and Cytotoxic Activities. Molecules.

[41] Lombardo D., Calandra P., Kiselev M.A. (2020). Structural Characterization of Biomaterials by Means of Small Angle X-Rays and Neutron Scattering (SAXS and SANS), and Light Scattering Experiments. Molecules.

[42] Grünewald T.A., Lassenberger A., Van Oostrum P.D.J., Rennhofer H., Zirbs R., Capone B., Vonderhaid I., Amenitsch H., Lichtenegger H.C., Reimhult E. (2015). Core–Shell Structure of Monodisperse Poly(Ethylene Glycol)-Grafted Iron Oxide Nanoparticles Studied by Small-Angle X-Ray Scattering. Chem. Mater..

[43] Urimi D., Hellsing M., Mahmoudi N., Söderberg C., Widenbring R., Gedda L., Edwards K., Loftsson T., Schipper N. (2022). Structural Characterization Study of a Lipid Nanocapsule Formulation Intended for Drug Delivery Applications Using Small-Angle Scattering Techniques. Mol. Pharm..

[44] Tian Y., Li L., Han H., Wang W., Wang Y., Ye Z., Guo X. (2016). Modification of Spherical Polyelectrolyte Brushes by Layer-by-Layer Self-Assembly as Observed by Small Angle X-Ray Scattering. Polymers.

[45] Riske K.A., Barroso R.P., Vequi-Suplicy C.C., Germano R., Henriques V.B., Lamy M.T. (2009). Lipid Bilayer Pre-Transition as the Beginning of the Melting Process. Biochim. Biophys. Acta (BBA)-Biomembr..

[46] Vist M.R., Davis J.H. (1990). Phase Equilibria of Cholesterol/Dipalmitoylphosphatidylcholine Mixtures: Deuterium Nuclear Magnetic Resonance and Differential Scanning Calorimetry. Biochemistry.

[47] Guimarães D., Cavaco-Paulo A., Nogueira E. (2021). Design of Liposomes as Drug Delivery System for Therapeutic Applications. Int. J. Pharm..

[48] Shariare M.H., Khan M.A., Al-Masum A., Khan J.H., Uddin J., Kazi M. (2022). Development of Stable Liposomal Drug Delivery System of Thymoquinone and Its In Vitro Anticancer Studies Using Breast Cancer and Cervical Cancer Cell Lines. Molecules.

[49] Giatrellis S., Nounesis G. (2011). Nucleic Acid-Lipid Membrane Interactions Studied by DSC. J. Pharm. Bioallied Sci..

[50] Lutta A., Martínez-Navarrete M., Tollemeto M., Andresen A.K., Pedersen G.K., Guillot A.J., Zhang Z., Grohganz H., Melero A., Schmidt S.T. (2026). The Role of Pectin in Optimizing Cationic Liposomes for Oral Delivery. ACS Appl. Bio Mater..

[51] Peppas N.A., Sahlin J.J. (1989). A Simple Equation for the Description of Solute Release. III. Coupling of Diffusion and Relaxation. Int. J. Pharm..

[52] Ritger P.L., Peppas N.A. (1987). A Simple Equation for Description of Solute Release I. Fickian and Non-Fickian Release from Non-Swellable Devices in the Form of Slabs, Spheres, Cylinders or Discs. J. Control. Release.

[53] Taciak B., Białasek M., Braniewska A., Sas Z., Sawicka P., Kiraga Ł., Rygiel T., Król M. (2018). Evaluation of Phenotypic and Functional Stability of RAW 264.7 Cell Line through Serial Passages. PLoS ONE.

[54] Chernomordik L.V., Kozlov M.M. (2008). Mechanics of Membrane Fusion. Nat. Struct. Mol. Biol..

[55] Kozlov M.M., McMahon H.T., Chernomordik L.V. (2010). Protein-Driven Membrane Stresses in Fusion and Fission. Trends Biochem. Sci..

[56] Caracciolo G. (2015). Liposome–Protein Corona in a Physiological Environment: Challenges and Opportunities for Targeted Delivery of Nanomedicines. Nanomed. Nanotechnol. Biol. Med..

[57] Engelberg S., Modrejewski J., Walter J.G., Livney Y.D., Assaraf Y.G. (2018). Cancer Cell-Selective, Clathrin-Mediated Endocytosis of Aptamer Decorated Nanoparticles. Oncotarget.

[58] Behzadi S., Serpooshan V., Tao W., Hamaly M.A., Alkawareek M.Y., Dreaden E.C., Brown D., Alkilany A.M., Farokhzad O.C., Mahmoudi M. (2017). Cellular Uptake of Nanoparticles: Journey inside the Cell. Chem. Soc. Rev..

[59] Harish V., Haque E., Śmiech M., Taniguchi H., Jamieson S., Tewari D., Bishayee A. (2021). Xanthohumol for Human Malignancies: Chemistry, Pharmacokinetics and Molecular Targets. Int. J. Mol. Sci..

[60] Gottesman M.M., Fojo T., Bates S.E. (2002). Multidrug Resistance in Cancer: Role of ATP–Dependent Transporters. Nat. Rev. Cancer.

[61] Bai X., Smith Z., Wang Y., Butterworth S., Tirella A. (2022). Sustained Drug Release from Smart Nanoparticles in Cancer Therapy: A Comprehensive Review. Micromachines.

