# Supplementary material for: Development of Pectin-Coated Cationic Liposomes for Sustained Drug Release, Enhanced Serum Stability and Reduced Macrophage Uptake
Source: Pharmaceutics. 2026 Oct 8;18(10):1275. doi: 10.3390/pharmaceutics18101275 (PMC13708220; doi:10.3390/pharmaceutics18101275)
Supplement: Supplementary file 1 [file pharmaceutics-18-01275-s001.zip › pharmaceutics-4520146-supplementary.pdf]

Supplementary materials

Development of Pectin-Coated Cationic Liposomes for Sustained Drug Release, Enhanced Serum Stability and Reduced Macrophage Uptake

Plamen Simeonov [1,2], Velislava Todorova [2,3], Tsvetelina Batsalova [4], Balik Dzhambazov [4], Stanislava Ivanova [2,3] and Plamen Katsarov [1,2,*]

[1] Department of Pharmaceutical Technology and Biopharmacy, Faculty of Pharmacy, Medical University of Plovdiv, 15A Vasil Aprilov blvd, 4002 Plovdiv, Bulgaria; plamen.simeonov@mu-plovdiv.bg

[2] Research Institute at Medical University of Plovdiv (RIMU), 4002 Plovdiv, Bulgaria; velislava.todorova@mu-plovdiv.bg (V.T.); stanislava.ivanova@mu-plovdiv.bg (S.I.)

[3] Department of Pharmacognosy and Pharmaceutical Chemistry, Faculty of Pharmacy, Medical University of Plovdiv, 15A Vasil Aprilov blvd, 4002 Plovdiv, Bulgaria

[4] Department of Developmental Biology, Faculty of Biology, Paisii Hilendarski University of Plovdiv, 24 Tsar Assen Str., 4000 Plovdiv, Bulgaria; tsvetelina@uni-plovdiv.bg (T.B.); balik@uni-plovdiv.bg (B.D.)

* Correspondence: plamen.katsarov@mu-plovdiv.bg

Table S1. Response table for Signal-to-Noise ratios for the average size model.

Table S2. Statistical data for the effect of pectin coating parameters on the average particle size.

Table S3. Response table for Signal-to-Noise ratios for the ζ-potential model.

Table S4. Statistical data for the effect of pectin coating parameters on the ζ-potential.

Table S5. Response table for Signal-to-Noise ratios for the polydispersity index model.

Table S6. Statistical for the effect of pectin coating parameters on the PDI.

Table S7. Thermodynamic parameters of pectin binding to xanthohumol-loaded cationic liposomes determined by isothermal titration calorimetry at 25°C.

Table S8. R2 - values of the fitted mathematical models for xanthohumol release profile.

Figure S1. IFT fit of SAXS scattering data and pair distance distribution functions p(r) for uncoated (A, B) and pectin-coated (C, D) liposomes.

Figure S2. In vitro release profiles of xanthohumol from unmodified and pectin-modified liposomes at pH 6.5.

Table S1. Response table for Signal-to-Noise ratios for the average size model

Level	Polysaccharide concentration	$CaCL_2$	Incubation time
1	-49,35	-49,56	-51,22
2	-49,75	-50,93	-50,51
3	-52,68	-51,29	-50,04
Delta	3,34	1,74	1,18
Rank	1	2	3

Smaller is better

Table S2. Statistical data for the effect of pectin coating parameters on the average particle size.

Term	Influence on the Average size				
	Coef	T-Value	VIF	F-Value	*p*-Value
Constant	336.72	70.45			
Polysaccharide concentration	70.12	36.25	1.04	1313.72	<0.001
$CaCl_2$ concentration	34.51	17.84	1.00	318.15	<0.001
Incubation time	-27.36	-14.14	1.04	200.04	<0.001
Polysaccharide conc.xPolysaccharide conc.	34.70	9.04	1.04	81.71	<0.001
$CaCl_2$ conc. x $CaCl_2$ conc.	-27.96	-8.23	1.00	67.66	<0.001
Incubation time x Incubation time	13.13	3.42	1.04	11.70	0.003

Table S3. Response table for Signal-to-Noise ratios for the ζ-potential model.

Level	Polysaccharide concentration	$CaCL_2$	Incubation time
1	24,27	28,38	24,45
2	24,04	22,83	24,11
3	23,81	20,91	23,56
Delta	0,46	7,47	0,90
Rank	3	1	2

Larger is better

Table S4. Statistical data for the effect of pectin coating parameters on the ζ-potential.

Term	Influence on the ζ-potential				
	Coef	T-Value	VIF	F-Value	*p*-Value
Constant	12.13	14.46			
Polysaccharide concentration	-0.97	-2.87	1.04	8.23	0.009
$CaCl_2$ concentration	-7.66	-22.55	1.00	508.41	<0.001
Incubation time	-1.30	-3.82	1.04	14.60	0.001
Polysaccharide conc. x Polysaccharide conc.	0.38	0.56	1.04	0.31	0.584
$CaCl_2$ conc. x $CaCl_2$ conc.	5.83	9.77	1.00	95.55	<0.001
Incubation time x Incubation time	0.42	0.62	1.04	0.38	0.542

Table S5. Response table for Signal-to-Noise ratios for the polydispersity index model.

Level	Polysaccharide concentration	$CaCL_2$	Incubation time
1	14,01	14,08	11,25
2	13,71	13,25	14,08
3	11,74	12,12	14,14
Delta	2,27	1,96	2,89
Rank	2	3	1

Smaller is better

Table S6. Statistical for the effect of pectin coating parameters on the PDI.

Term	Influence on the Polydispersity index				
	**Coef**	**T-Value**	**VIF**	**F-Value**	***p*-Value**
Constant	0.183	10.11			
Polysaccharide concentration	0.032	4.34	1.04	18.80	<0.001
$CaCl_2$ concentration	0.025	3.42	1.00	11.72	0.003
Incubation time	-0.040	-5.48	1.04	30.00	<0.001
Polysaccharide conc. x Polysaccharide conc.	0.009	0.6	1.04	0.36	0.553
$CaCl_2$ conc. x $CaCl_2$ conc.	0.002	0.18	1.00	0.03	0.863
Incubation time x Incubation time	0.049	3.36	1.04	11.32	0.003

Table S7. Thermodynamic parameters of pectin binding to xanthohumol-loaded cationic liposomes determined by isothermal titration calorimetry at 25°C.

Parameter	Pectin -> Liposomes
Ka (M^{-1})	1.639×10^4
Kd (M)	6.102×10^{-5}
ΔH (kJ/mol)	−199.9
ΔG (kJ/mol)	−24.06
ΔS (J/mol·K)	−751.2
N	3 (pectin:liposome)

Table S8. R^2 - values of the fitted mathematical models for xanthohumol release profile.

Model	Unmodified liposomes	Pectin-modified liposomes
Zero-order	0.4154	0.4705
First-order	0.9104	0.6584
Higuchi	0.6706	0.6972
Hixon-Crowell	0.7827	0.5946
Baker-Lonsdale	0.8925	0.8557
Peppas - Sahlin	0.9926	0.9888

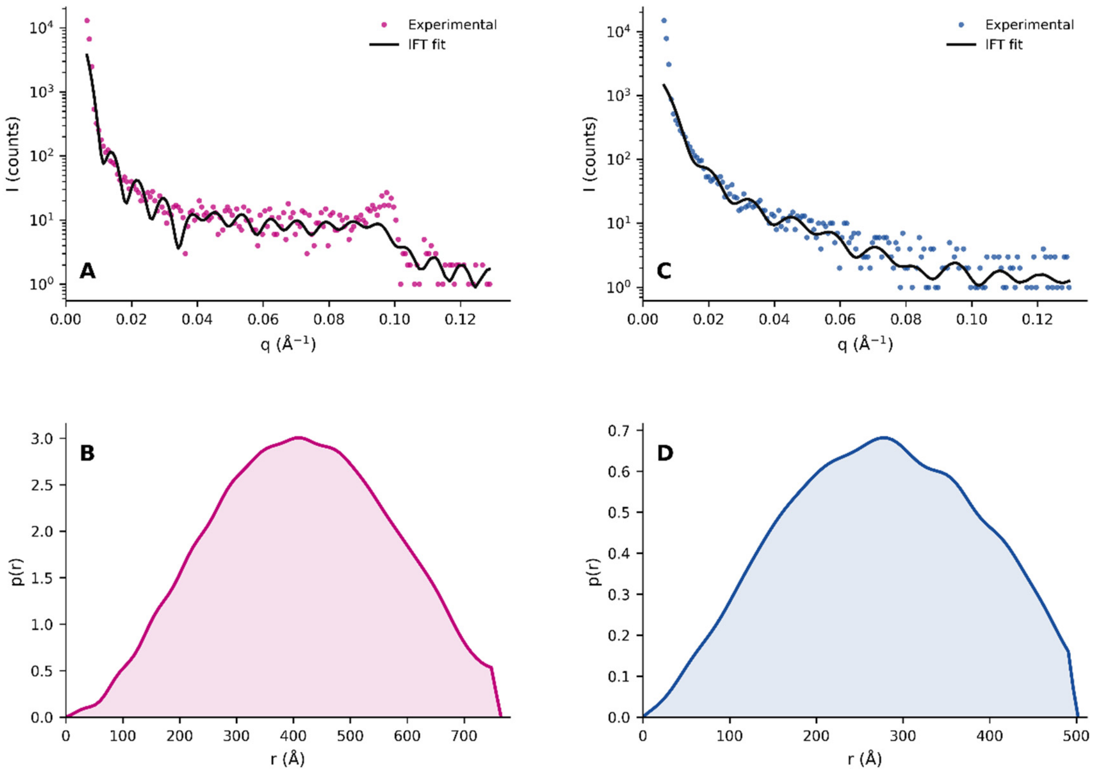

Figure S1. IFT fit of SAXS scattering data and pair distance distribution functions p(r) for uncoated (A, B) and pectin-coated (C, D) liposomes.

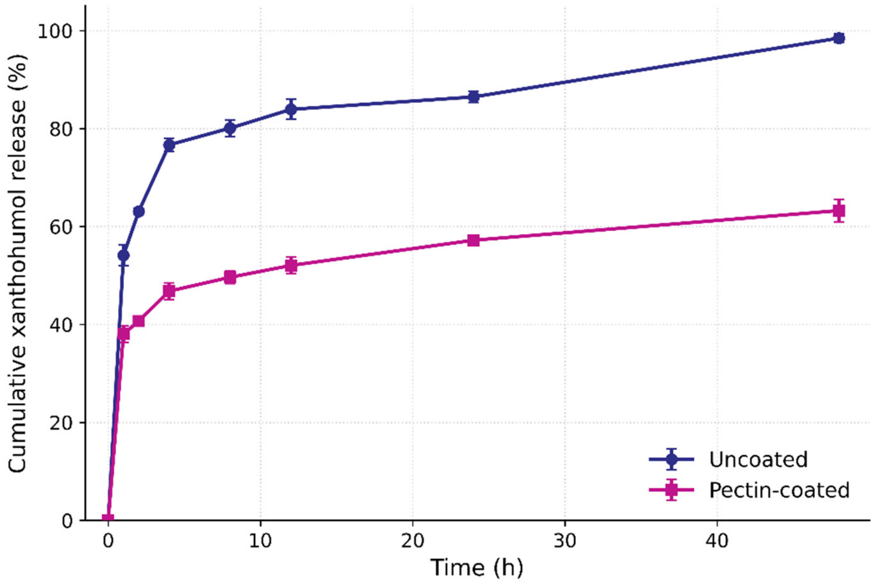

Figure S2. In vitro release profiles of xanthohumol from unmodified and pectin-modified liposomes at pH 6.5.
